# Supplementary material for: Gestational diabetes mellitus and offspring’s carotid intima–media thickness at birth: MySweetHeart Cohort study
Source: BMJ Open. 2022 Jul 26;12(7):e061649. doi: 10.1136/bmjopen-2022-061649 (PMC9330339; doi:10.1136/bmjopen-2022-061649)
Supplement: Supplementary data [file bmjopen-2022-061649supp001.pdf]

SUPPLEMENTARY MATERIAL

Gestational diabetes mellitus and offspring's carotid intima-media thickness at birth: *MySweetHeart* Cohort Study

Adina Mihaela Epure[1,2], Stefano Di Bernardo[3], Yvan Mivelaz[3], Sandrine Estoppey Younes[2], Arnaud Chiolero[1,4,5¶], Nicole Sekarski[3¶], on behalf of *MySweetHeart* Research Group

[1]Population Health Laboratory (#PopHealthLab), University of Fribourg, Fribourg, Switzerland;

[2]Department of Epidemiology and Health Services, Center for Primary Care and Public Health (UNISANTÉ), University of Lausanne, Lausanne, Switzerland;

[3]Paediatric Cardiology Unit, Woman-Mother-Child Department, Lausanne University Hospital (CHUV), Lausanne, Switzerland;

[4]Institute of Primary Health Care (BIHAM), University of Bern, Bern, Switzerland;

[5]School of Global and Population Health, McGill University, Montréal, Canada

¶ These authors contributed equally to this work.

Epure AM, *et al. BMJ Open* 2022; 12:e061649. doi: 10.1136/bmjopen-2022-061649

Table S1 The relationship of GDM and assignment or not to a lifestyle and psychosocial intervention with offspring's CIMT at birth.

	Mean (sd), mm	**Model 1 (n=200)**		**Model 2 (n=165)**		**Model 3 (n=165)**	
		Mean difference (95% CI), mm	p	Mean difference (95% CI), mm	p	Mean difference (95% CI), mm	p
Non-GDM	0.30 (0.04)	ref	ref	ref	ref	ref	ref
GDM, non-I	0.30 (0.04)	0.00 (-0.01 to 0.01)	0.94	0.00 (-0.02 to 0.02)	0.93	0.00 (-0.02 to 0.01)	0.91
GDM, I	0.30 (0.04)	0.00 (-0.01 to 0.01)	0.98	-0.01 (-0.03 to 0.01)	0.19	-0.01 (-0.02 to 0.01)	0.25

Estimates were obtained from linear regression models with the following specification: Model 1: unadjusted estimates; Model 2: estimates adjusted for maternal pre-pregnancy BMI, education, and tobacco smoking; offspring family history of diabetes and sex; Model 3: estimates adjusted for maternal pre-pregnancy BMI, education, and tobacco smoking; offspring family history of diabetes, sex, body surface area and age at CIMT assessment. The outcome variable (i.e., CIMT) was continuous. The exposure variable was categorical with 3 levels (Non-GDM/ GDM, non-I/ GDM, I; the reference category was Non-GDM). Similar results were obtained when Model 1 was run in the sample (n=165) with data on outcome, exposure, and all covariates included in Models 2 and 3 (GDM, non-I: 0.00 mm (95% CI: -0.01 to 0.01; p=0.99); GDM, I: -0.01 mm (95% CI: -0.02 to 0.01; p=0.29)). Abbreviations: CI, confidence interval; CIMT, carotid intima-media thickness; GDM, gestational diabetes mellitus; I, intervention; n, total number of participants; p, p-value; sd, standard deviation; ref, reference group.

Epure AM, *et al. BMJ Open* 2022; 12:e061649. doi: 10.1136/bmjopen-2022-061649

Fig. S1 Illustration of the relationship of GDM and assignment or not to a lifestyle and psychosocial intervention with offspring's CIMT through a forest plot.

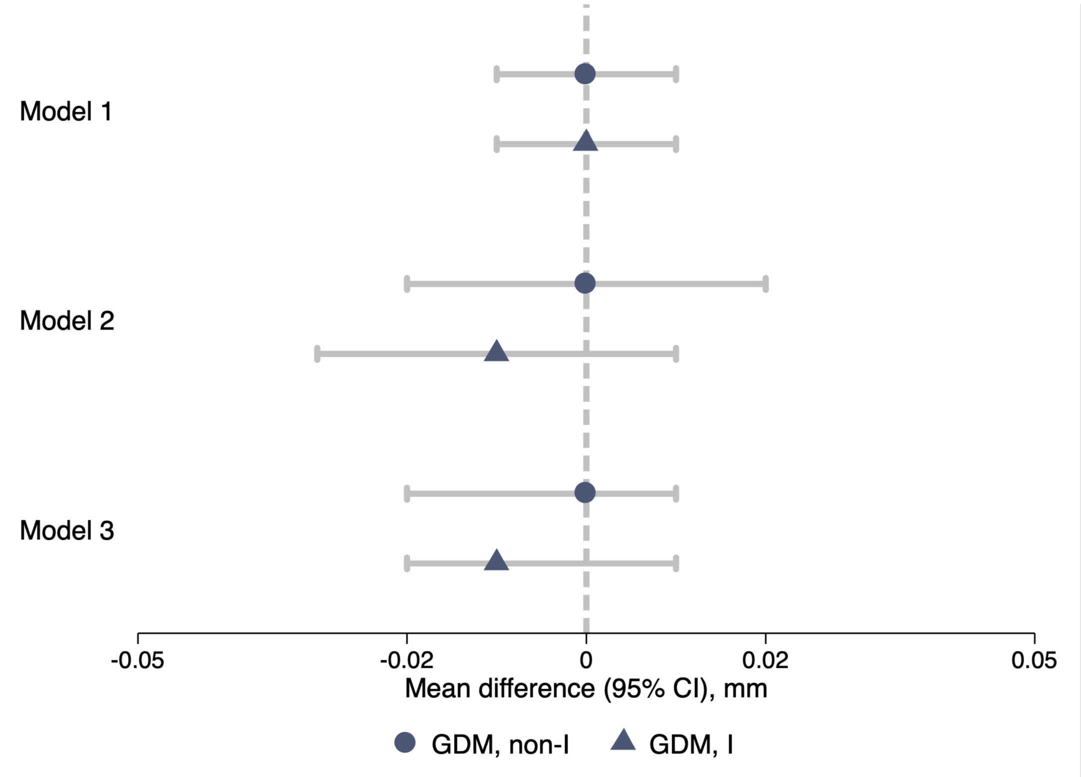

Figure legend The circles represent mean differences in CIMT between offspring of women with GDM assigned to no intervention (*GDM, non-I*) and offspring of women without GDM (*Non-GDM*). The triangles represent mean differences in CIMT between offspring of women with GDM assigned to a lifestyle and psychosocial intervention (*GDM, I*) and offspring of women without GDM (*Non-GDM*). The horizontal lines represent the 95% CIs. The plot was constructed using regression estimates and models presented in Table S1. Model specification: Model 1 is unadjusted, while Models 2 and 3 are adjusted for various factors as described in the methods and footnote of Table S1. Abbreviations: CI, confidence interval; CIMT, carotid intima-media thickness; GDM, gestational diabetes; I, intervention.

Epure AM, *et al. BMJ Open* 2022; 12:e061649. doi: 10.1136/bmjopen-2022-061649

Fig. S2 Dot plot of CIMT at birth by gestational diabetes mellitus (GDM) and assignment to a lifestyle and psychosocial intervention (I).

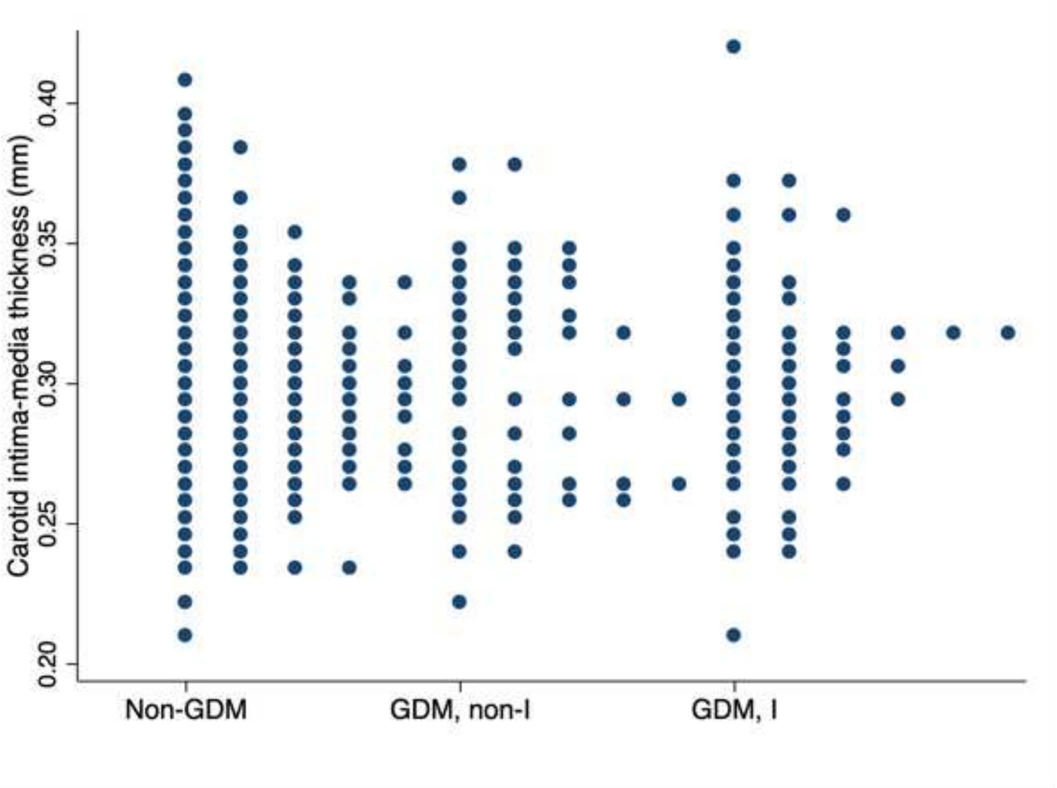

Figure legend This figure shows the distribution of CIMT in the offspring of women without GDM (*Non-GDM*) and the offspring of women with GDM who were assigned to no intervention (*GDM, non-I*) or to a lifestyle and psychosocial intervention (*GDM, I*) as part of their participation in the *MySweetHeart* Trial. Abbreviations: CIMT, carotid intima-media thickness; GDM, gestational diabetes mellitus; I, intervention.
