# Supplementary material for: Gestational diabetes mellitus and offspring’s carotid intima–media thickness at birth: MySweetHeart Cohort study
Source: BMJ Open. 2022 Jul 26;12(7):e061649. doi: 10.1136/bmjopen-2022-061649 (PMC9330339; doi:10.1136/bmjopen-2022-061649)
Supplement: Author's manuscript [file bmjopen-2022-061649.draft_revisions.pdf]

BMJ Open

BMJ Open is committed to open peer review. As part of this commitment we make the peer review history of every article we publish publicly available.

When an article is published we post the peer reviewers' comments and the authors' responses online. We also post the versions of the paper that were used during peer review. These are the versions that the peer review comments apply to.

The versions of the paper that follow are the versions that were submitted during the peer review process. They are not the versions of record or the final published versions. They should not be cited or distributed as the published version of this manuscript.

BMJ Open is an open access journal and the full, final, typeset and author-corrected version of record of the manuscript is available on our site with no access controls, subscription charges or pay-per-view fees (http://bmjopen.bmj.com).

If you have any questions on BMJ Open's open peer review process please email info.bmjopen@bmj.com

Gestational diabetes mellitus and offspring's carotid intima-media thickness at birth: MySweetHeart Cohort Study

Journal:	*BMJ Open*
Manuscript ID	bmjopen-2022-061649
Article Type:	Original research
Date Submitted by the Author:	31-Jan-2022

Complete List of Authors:	Epure, Adina Mihaela; University of Fribourg, Population Health Laboratory; University of Lausanne, Center for Primary Care and Public Health, Department of Epidemiology and Health Services<br>Di Bernardo, Stefano; Lausanne University Hospital, Paediatric Cardiology Unit, Department Woman-Mother-Child<br>Mivelaz, Yvan; Lausanne University Hospital, Paediatric Cardiology Unit, Department Woman-Mother-Child<br>Estoppey Younes, Sandrine; University of Lausanne, Center for Primary Care and Public Health, Department of Epidemiology and Health Services<br>Chiolero, Arnaud; University of Fribourg, Population Health Laboratory; University of Bern, Institute of Primary Health Care<br>Sekarski, Nicole; Lausanne University Hospital, Paediatric Cardiology Unit, Woman-Mother-Child Department

Keywords:	Cardiovascular imaging < RADIOLOGY & IMAGING, Paediatric cardiology < CARDIOLOGY, Diabetes in pregnancy < DIABETES & ENDOCRINOLOGY, NEONATOLOGY, Cardiac Epidemiology < CARDIOLOGY

SCHOLARONE™ Manuscripts

I, the Submitting Author has the right to grant and does grant on behalf of all authors of the Work (as defined in the below author licence), an exclusive licence and/or a non-exclusive licence for contributions from authors who are: i) UK Crown employees; ii) where BMJ has agreed a CC-BY licence shall apply, and/or iii) in accordance with the terms applicable for US Federal Government officers or employees acting as part of their official duties; on a worldwide, perpetual, irrevocable, royalty-free basis to BMJ Publishing Group Ltd ("BMJ") its licensees and where the relevant Journal is co-owned by BMJ to the co-owners of the Journal, to publish the Work in this journal and any other BMJ products and to exploit all rights, as set out in our licence.

The Submitting Author accepts and understands that any supply made under these terms is made by BMJ to the Submitting Author unless you are acting as an employee on behalf of your employer or a postgraduate student of an affiliated institution which is paying any applicable article publishing charge ("APC") for Open Access articles. Where the Submitting Author wishes to make the Work available on an Open Access basis (and intends to pay the relevant APC), the terms of reuse of such Open Access shall be governed by a Creative Commons licence – details of these licences and which Creative Commons licence will apply to this Work are set out in our licence referred to above.

Other than as permitted in any relevant BMJ Author's Self Archiving Policies, I confirm this Work has not been accepted for publication elsewhere, is not being considered for publication elsewhere and does not duplicate material already published. I confirm all authors consent to publication of this Work and authorise the granting of this licence.

For peer review only

Gestational diabetes mellitus and offspring's carotid intima-media thickness at birth: *MySweetHeart* Cohort Study

Adina Mihaela Epure[1,2], Stefano Di Bernardo[3], Yvan Mivelaz[3], Sandrine Estoppey Younes[2], Arnaud Chiolero[1,4,5¶], Nicole Sekarski[3¶*], on behalf of *MySweetHeart* Research Group

[1]Population Health Laboratory (#PopHealthLab), University of Fribourg, Fribourg, Switzerland;

[2]Department of Epidemiology and Health Services, Center for Primary Care and Public Health (UNISANTÉ), University of Lausanne, Lausanne, Switzerland;

[3]Paediatric Cardiology Unit, Woman-Mother-Child Department, Lausanne University Hospital (CHUV), Lausanne, Switzerland;

[4]Institute of Primary Health Care (BIHAM), University of Bern, Bern, Switzerland;

[5]School of Global and Population Health, McGill University, Montréal, Canada

¶ These authors contributed equally to this work.

* **Corresponding author:** Prof Nicole Sekarski, nicole.sekarski@chuv.ch, Lausanne University Hospital (CHUV), 46 Route du Bugnon, Lausanne, 1011, Switzerland

Word count (excluding title page, abstract and article summary, references, figure legends and tables): 2'893

For peer review only - http://bmjopen.bmj.com/site/about/guidelines.xhtml

ABSTRACT

Objective Hyperglycemia during pregnancy is associated with cardiometabolic risks for the mother and the offspring. Mothers with gestational diabetes mellitus (GDM) have signs of subclinical atherosclerosis, including increased carotid intima-media thickness (CIMT). We assessed whether GDM is associated with increased CIMT in the offspring at birth.

Design and setting *MySweetHeart* Cohort is a prospective cohort study conducted in Switzerland.

Participants, exposure and outcome measures This work included pregnant women with and without GDM at 24 to 32 weeks of gestation and their singleton live-born offspring with data on the primary outcome of CIMT. GDM was diagnosed based on the criteria of the International Association of Diabetes and Pregnancy Study Groups. Offspring's CIMT was measured by ultrasonography after birth (range: 1 to 19 days).

Results Data on CIMT were available for 99 offspring of women without GDM and 101 offspring of women with GDM. Maternal age ranged from 18 to 47 years. Some 16% of women with GDM and 6% of women without GDM were obese. Smoking during pregnancy was more frequent among women with GDM (18%) than among those without GDM (4%). Neonatal characteristics were comparable between the 2 groups. The difference in CIMT between offspring of women with and without GDM was of 0.00 mm (95% CI: -0.01 to 0.01; p=0.96) and remained similar upon adjustment for potential confounding factors, such as maternal pre-pregnancy BMI, maternal education,

For peer review only

smoking during pregnancy, family history of diabetes, as well as offspring's sex, age, and body surface area (0.00 mm (95% CI: -0.02 to 0.01; p=0.45)).

Conclusions We found no evidence of increased CIMT in neonates exposed to GDM. A longer-term follow-up that includes additional vascular measures, such as endothelial function or arterial stiffness, may shed further light on the cardiovascular health trajectories in children born to mothers with GDM.

Registration ClinicalTrials.gov (NCT02872974)

Keywords gestational diabetes; carotid intima-media thickness; cardiovascular prevention; child; neonate

List of abbreviations BMI, body mass index; CIMT, carotid intima-media thickness; CVD, cardiovascular disease; CHUV, Lausanne University Hospital; DOHaD, developmental origins of health and disease; FPG, fasting plasma glucose; GDM, gestational diabetes mellitus; HbA1c, glycated hemoglobin; IADPSG, International Association of Diabetes and Pregnancy Study Groups; I, intervention; OGTT, oral glucose tolerance test.

For peer review only

Strengths and limitations of this study

- One important strength of this study is represented by its prospective design and the enrollment of participants at the time of gestational diabetes diagnosis.
- Carotid intima-media thickness was measured in non-sedated neonates by experienced pediatric cardiologists using automated methods with manual tracing adjustment, in accordance with published guidelines.
- Limitations of this study include the relatively small sample size, the possibility of residual confounding and the limited generalizability.

For peer review only

For peer review only - http://bmjopen.bmj.com/site/about/guidelines.xhtml

INTRODUCTION

Gestational diabetes mellitus (GDM) is a state of hyperglycemia with onset or first recognition during pregnancy.[1-3] The prevalence of hyperglycemia during pregnancy has increased in recent decades, being estimated at 16% worldwide in 2019, with 84% of cases due to GDM.[4] GDM is associated with long-term metabolic consequences for both the mother and the offspring, such as type 2 diabetes and obesity.[5] Women with GDM also have subclinical atherosclerosis and an increased risk for cardiovascular disease (CVD) later in life.[6,7] However, little is known about the cardiovascular risk of their offspring.

CIMT is a surrogate marker of atherosclerosis, which has been shown to be increased in children exposed to risk factors in the first 1000 days of life, such as poor fetal growth,[8] as well as in children with type 1 diabetes.[9] From a developmental origins of health and disease (DOHaD) perspective,[10] exposure to adverse experiences in early life may produce lifelong adaptations in the organs' structure and function and may program the risk for CVD. For instance, a systematic review and meta-analysis showed that GDM was associated with a higher systolic blood pressure in childhood.[11] Whether GDM has an impact on children's CIMT is not clearly established. The evidence is scarce notably in the very young children although CIMT measurement is feasible from birth and could help discern between changes that occur before or after birth.[12] To fill this gap, we conducted *MySweetHeart Cohort* study to assess the early life cardiovascular consequences of GDM.[13] Herein, we evaluated CIMT at birth in offspring of mothers with and without GDM.

For peer review only - http://bmjopen.bmj.com/site/about/guidelines.xhtml

METHODS

Study design and setting

MySweetHeart Cohort is a prospective cohort study conducted at the Lausanne University Hospital (CHUV), Switzerland. The study has been registered with ClinicalTrials.gov (clinicaltrials.gov/ct2/show/NCT02872974) and the study protocol has been published.[13] Ethical approval was granted by the Ethics Committee for Human Research of the Canton de Vaud (study number 2016-00745).

Study population

This cohort included pregnant women between 24 and 32 weeks of gestation, with and without GDM. Other inclusion criteria were age 18 years or more and understanding French or English. The exclusion criteria were pre-existing diabetes mellitus, strict bed rest, or severe mental disorders. To facilitate recruitment and share resources, a collaboration was established with *MySweetHeart Trial*,[14] a randomized controlled trial assessing the effect of a lifestyle and psychosocial intervention on cardiometabolic outcomes of women with GDM and their offspring. As such, women with GDM were invited to contribute to both studies. Participating women with and without GDM were included in the current analysis if CIMT data for their live-born singleton neonates were available. All families gave a signed informed consent for use of their data.

Data collection

GDM screening

Pregnant women screened at the prenatal care clinic of the CHUV had a fasting plasma glucose (FPG) test between 24 and 28 weeks of gestation and GDM was diagnosed if the test result was ≥ 5.1 mmol/L.[13] If FPG was < 5.1 mmol/L, but ≥ 4.4 mmol/L,

For peer review only - http://bmjopen.bmj.com/site/about/guidelines.xhtml

women had a 2-hour 75 g oral glucose tolerance test (OGTT) and GDM was diagnosed based on the criteria of the International Association of Diabetes and Pregnancy Study Groups (IADPSG).[15] Pregnant women screened by external obstetricians in the Canton of Vaud underwent the same procedure or directly a 2-hour 75 g OGTT.

Carotid ultrasound and CIMT measurement

A carotid ultrasound assessment was performed between 1 and 7 days of life in the majority of neonates (n=191). A small share (n=9) had the exam between 8 and 19 days of life due to organizational and logistical constraints. Parents were told to feed and burp their offspring ahead of the carotid ultrasound to make them more relaxed. Feeding or administration of a 30% glucose solution were used to comfort the neonates if they became agitated during the exam. The exam took place in a dark and quiet room and a cloth was placed under the neonates' shoulders to facilitate the extension of the neck.

Ultrasound image acquisition and analysis were performed by 2 experienced pediatric cardiologists who were blinded to the maternal glycemic status. Images were acquired in B-mode with no harmonics, sonoCT, dynamic range of 60dB, at a frame rate of 100-120Hz, with a depth of 1-2 cm. The right and left carotid arteries were scanned using a Philips EPIC echocardiograph (Philips Medical, Netherlands) with a L 15-7 MHz high-resolution linear array transducer, according to the American Heart Association's recommendations for standard assessment of subclinical atherosclerosis in children and adolescents.[16] Each observer recorded three consecutive 3-second cine loops from 2 different angles on each side, which were stored as native DICOM for subsequent offline analyses (QLab, Philips Medical, Netherlands). Whenever image

For peer review only - http://bmjopen.bmj.com/site/about/guidelines.xhtml

quality was optimal enough, 6 right and 6 left frames were selected and, for each, the maximal IMT of the common carotid artery far wall was measured. Measurements were performed over a 1-cm region of interest proximal to the carotid bulb, on or closest to the R-wave of the electrocardiogram, using a semi-automated edge detection software with manual tracing adjustment when needed. The mean of 12 maximal CIMT measurements was used in the analysis for the majority of neonates (n=170). Two neonates had only one measurement available, whereas the rest had between 2 and 11 measurements that were averaged. A good interobserver reliability (coefficient of variation=5.9%) for measurements in non-sedated infants was proven in our laboratory previously.[12]

Other sample characteristics

Data on maternal characteristics (age, country of origin, education, smoking during pregnancy, pre-pregnancy weight and height, or parity) and family history of diabetes were record-based or self-reported by the mother at a researcher-administered interview upon inclusion in the study. Smoking during pregnancy was defined as a mother who was an active tobacco smoker at study baseline, i.e., between 24 and 32 weeks of gestation. A maternal blood sampling was also performed at baseline and glycated hemoglobin (HbA1c) was measured. Pre-pregnancy body mass index (BMI) was computed by dividing the pre-pregnancy weight (kg) by the squared height (m^2). Delivery data such as newborn sex, anthropometry, gestational age, or mode of delivery were obtained from the medical records. Neonatal weight, length and blood pressure were measured by the study team at the time of the carotid ultrasound. Body surface area (m^2) was computed using the Mosteller equation.[17] One systolic and diastolic blood pressure measurement was taken from the right upper arm, in a supine

For peer review only - http://bmjopen.bmj.com/site/about/guidelines.xhtml

position, using a clinically validated and regularly calibrated oscillometric sphygmomanometer (Accutorr Plus; Datascope, Paramus, New Jersey, USA) with neonate cuffs.

Data analysis

Descriptive statistics on study participants are reported as percentages (%) or as mean, standard deviation, minimum and maximum values. The relationship of GDM with CIMT was evaluated by a set of linear regression models with and without adjustment for potential confounders, i.e., baseline covariates associated with metabolic and cardiovascular risks, offspring's sex, and anthropometry at CIMT assessment. Potential confounders were maternal pre-pregnancy BMI, maternal education (university/no university), smoking during pregnancy (yes/no), and family history of diabetes (yes/no). The variable family history of diabetes summarized disease occurrence in a 1st degree relative of the mother, 1st degree relative of the father or in the father himself and assumed missing data in any of these variables as no history of diabetes unless values for all 3 variables were missing. To account for differences in body size,[18,19] we adjusted for body surface area and age at CIMT assessment. All statistical analyses were performed in Stata 16 (Stata Corporation, Texas, USA).

RESULTS

Characteristics of study participants

Data collection started in September 2016 and ended in October 2020. A total of 137 participants without GDM exposure and 212 participants with GDM exposure were recruited in the study. Some 101 neonates without GDM exposure and 117 neonates

For peer review only - http://bmjopen.bmj.com/site/about/guidelines.xhtml

with GDM exposure attended the cardiovascular follow-up visit early after birth. Of these, 200 singleton neonates born at more than 36 weeks of gestation (non-GDM: n=99; GDM: n=101) had CIMT measurements and constitute the analytic sample for the current analysis.

Family and neonatal characteristics of study participants are presented in Table 1. The maternal characteristics were generally comparable between the non-GDM and GDM groups. The majority of women were non-Swiss and their age ranged from 18 to 47 years. Approximately half of the women in each group had a high level of education and no previous deliveries. More women with GDM (16%) were obese (pre-pregnancy BMI≥ 30 kg/m^2) compared to women without GDM (6%). Smoking during pregnancy was more frequent among women with GDM (18%) than among those without GDM (4%). Offspring of women with and without GDM had similar neonatal characteristics, such as sex, gestational age, birth weight, length, or blood pressure. The majority were born at term, i.e., between 37 and 41 weeks (GDM: 96%; non-GDM: 98%) and a small share had macrosomia, i.e., a birth weight higher than 4’000 g (GDM: 6%; non-GDM: 5%). Offspring of women with GDM (46%) had a higher frequency of family history of diabetes compared to their non-GDM counterparts (24%).

For peer review only

For peer review only - http://bmjopen.bmj.com/site/about/guidelines.xhtml

Table 1 Characteristics of study participants by GDM exposure.

	Non-GDM[a] (n=99)				GDM[b] (n=101)			
	**mean or %**	**sd**	**min**	**max**	**mean or %**	**sd**	**min**	**max**
**MATERNAL**								
Age (years)	33	5	18	44	33	5	21	47
Swiss origin (%)	24				33			
University education (%)	60				55			
Primiparous (%)	55				48			
Smoking during pregnancy (%)	4				18			
Pre-pregnancy obesity (BMI ≥ 30 kg/m2) (%)	6				16			
HbA1C (%)	4.9	0.3	4.2	5.7	5.3	0.3	4.7	7.2
**NEONATAL**								
Male (%)	52				53			
Cesarean section (%)	22				32			
Term birth (37 to 41 weeks) (%)	98				96			
Birth weight (g)	3'352	425	2'190	4'190	3'357	442	2'220	4'340
Macrosomia (Birth weight > 4'000 g) (%)	5				6			
Length (cm)	50	2	45	54	50	2	45	56
Body surface area (m^2)	0.21	0.02	0.16	0.25	0.21	0.02	0.17	0.26
Systolic BP (mmHg)	78	9	60	101	78	10	60	111
Diastolic BP (mmHg)	47	8	30	66	48	10	28	90
Family history of diabetes (%)	24				46			

Abbreviations: BMI, body mass index; BP, blood pressure; GDM, gestational diabetes mellitus; HbA1c, glycated hemoglobin n, total number of participants; max, maximum; min, minimum; sd, standard deviation.

[a] Non-GDM: Missing values for swiss origin (n=1), university education (n=2), pre-pregnancy obesity (n=1), HbA1c (n=13), cesarean section (n=4), term birth (n=10), systolic BP (n=1), diastolic BP (n=1); family history of diabetes (n=1).
[b] GDM: Missing values for age (n=3), swiss origin (n=3), university education (n=18), primiparous (n=3), smoking (n=5), pre-pregnancy obesity (n=4), HbA1c (n=5), male (n=16), cesarean section (n=6), term birth (n=16), birth weight (n=16); family history of diabetes (n=4).

For peer review only

GDM and CIMT at birth

The distribution of CIMT values is presented in Fig. 1 and Fig. 2. CIMT ranged from 0.21 to 0.42 mm, with a mean CIMT of 0.30 mm (sd 0.04) overall and in each of the studied groups (Table 2, Table S1 in Supplementary Material).

Fig. 1 Histograms of CIMT at birth, overall and by GDM exposure.

Figure legend This figure shows the distribution of CIMT values in our sample, overall (n=200) and by GDM exposure (Non-GDM: n=99; GDM: n=101). The black line represents the kernel density estimate. Abbreviations: CIMT, carotid intima-media thickness; GDM, gestational diabetes mellitus.

Fig. 2 Box plots of CIMT at birth by GDM exposure and assignment to a lifestyle and psychosocial intervention.

Figure legend This figure shows the distribution of CIMT in the offspring of women without GDM (*Non-GDM*; n=99) and the offspring of women with GDM who were assigned to no intervention (*GDM, non-I*; n=48) or to a lifestyle and psychosocial intervention (*GDM, I*; n=53) as part of their participation in the *MySweetHeart* Trial. Abbreviations: CIMT, carotid intima-media thickness; GDM, gestational diabetes mellitus; I, intervention.

For peer review only

Table 2 The relationship of GDM with offspring's CIMT at birth.

	Mean (SD), mm	**Model 1 (n=200)**		**Model 2 (n=165)**		**Model 3 (n=165)**	
		Difference (95% CI), mm	p	Difference (95% CI), mm	p	Difference (95% CI), mm	p
Non-GDM	0.30 (0.04)	ref	ref	ref	ref	ref	ref
GDM	0.30 (0.04)	0.00 (-0.01 to 0.01)	0.96	0.00 (-0.02 to 0.01)	0.47	0.00 (-0.02 to 0.01)	0.45

Model 1: unadjusted estimates.

Model 2: estimates adjusted for maternal pre-pregnancy BMI, education, and tobacco smoking; offspring family history of diabetes and sex.

Model 3: estimates adjusted for maternal pre-pregnancy BMI, education, and tobacco smoking; offspring family history of diabetes, sex, body surface area and age at CIMT assessment.

Abbreviations: CI, confidence interval; CIMT, carotid intima-media thickness; GDM, gestational diabetes mellitus; n, total number of participants; p, p-value; sd, standard deviation; ref, reference group.

Note: Similar results were obtained when Model 1 was run in the sample (n=165) with data on outcome, exposure, and all covariates included in Models 2 and 3 (GDM: 0.00 mm (95% CI: -0.02 to 0.01; p=0.54)).

For peer review only

For peer review only - http://bmjopen.bmj.com/site/about/guidelines.xhtml

The relationship of GDM with offspring's CIMT early after birth is presented in Table 2. In the unadjusted analysis (Model 1), the difference in CIMT between offspring of women with and without GDM was 0.00 mm (95% CI -0.01 to 0.01; p=0.96). Adjustment for offspring sex and potential confounding factors (Model 2), as well as for offspring's body surface area and age at CIMT assessment (Model 3), resulted in a difference of 0 mm (95% CI -0.02 to 0.01; p=0.45). When exposure to GDM was analyzed separately for offspring whose mothers were assigned or not to a lifestyle and psychosocial intervention as part of their participation in *MySweetHeart* Trial, results were similar to those presented above (Table S1 in Supplementary Material).

DISCUSSION

Summary of findings and comparison with other studies

Our goal was to assess the relationship of GDM with neonatal CIMT. We found no evidence of an increased CIMT in neonates born to women with GDM as compared to those born to women without GDM. Our findings are in line with other studies that evaluated CIMT after intrauterine exposure to maternal hyperglycemia. A recent meta-analysis pooled data from 3 studies and reported no clear evidence of increased CIMT in children exposed to maternal hyperglycemia compared to those not exposed (pooled standardized mean difference (SMD): 0.08 (95% CI -0.16 to 0.33)).[8] Two of these studies included 6-year and 8-year children, respectively, and found no difference in CIMT after exposure to GDM (SMD 0.00 (95% CI -0.28 to 0.28) at 6 years and 0.00 (95% CI -0.41 to 0.41) at 8 years).[8,20,21] The third study included neonates and found a slightly higher CIMT among those exposed to diabetes (SMD 0.46 (95% CI -0.07 to 1.00)),[8,22] but the imprecision around the estimated difference was high, the

For peer review only - http://bmjopen.bmj.com/site/about/guidelines.xhtml

study had a very small sample size (n=55) and the authors did not specify whether they included women with pre-gestational or gestational diabetes.[22]

Strengths and limitations

A major strength of this study is its prospective design. Enrollment of study participants and collection of baseline characteristics took place close to the moment of GDM diagnosis and ahead of the CIMT outcome assessment. This implies that the choice of participation in the study is unlikely to be related to both the exposure and the outcome, which makes selection bias due to enrollment unlikely. Further, GDM was diagnosed using the new criteria of the IADPG. These criteria were derived based on the risk of adverse neonatal outcomes, such as birth weight, cord blood C-peptide levels, or percent infant body fat > 90th percentile.[15] They were endorsed by the World Health Organization along with several other bodies to achieve a universal consensus for GDM diagnosis and increase comparability of the evidence.[23,24] Another strength is the assessment of ultrasound CIMT using automated methods with manual tracing adjustment, in accordance with the current guidelines in children.[16,25] The semi-automated methods are associated with a lower interoperator variability and high reliability,[16,25] including in infants, as it was previously proved in our laboratory.[12]

This study has some limitations. Firstly, our results have limited generalizability, as we used a convenient sample of pregnant women recruited from health care facilities in Switzerland. Secondly, the GDM glucose screening test (FPG or 75-g OGTT) varied between participants. This is because our hospital used a 2-step targeted approach for identifying women with GDM. While the 2-step approach is practical and more acceptable to patients,[26] it may be related to a lower likelihood of diagnosing GDM

For peer review only

For peer review only - http://bmjopen.bmj.com/site/about/guidelines.xhtml

compared to a one-step universal screening based on a 75-g OGTT.[27] On the other hand, the IADGSP criteria, which have a lower threshold for a positive FPG test (≥5.1 mmol/L) compared to other guidelines,[23] may identify as having GDM women who are at low absolute risk for fetal and pregnancy complications and, thus, overdiagnose GDM in some populations.[28,29] Therefore, misclassification of the exposure cannot be excluded and our estimates of association might be biased, maybe underestimated. Additionally, women with GDM participated in *MySweetHeart* Trial and approximately half of them were assigned to a lifestyle and psychosocial intervention with the aim of improving their cardiometabolic outcomes. Although this intervention could have also modified the association of GDM with CIMT, this seems not likely, as mean CIMT values were very similar in offspring of women with GDM who participated in the intervention and the control arms of the trial. Thirdly, CIMT was assessed using conventional high-resolution ultrasound frequencies (< 15 MHz), which tend to overestimate the arterial thickness in the young children when compared to very high-resolution ultrasound systems (25 to 55 MHz).[30,31] Measurement error in CIMT cannot be excluded, but systematic differences between the two groups are unlikely because the outcome assessors were blinded to the glycemic status of the mothers. Fourthly, while we adjusted for key confounders at the analysis stage, there is a possibility of residual confounding due to the relatively small sample size and some imprecision in the measurement of confounder variables, especially in those self-reported. Lastly, our study was limited to CIMT, which is a measure of arterial structure. In fact, changes in the vessel function might occur earlier than changes in the vessel structure, therefore, a combination of vascular measures would be needed for a clearer view on the cardiovascular status of children exposed to adverse experiences in early life. However, certain techniques to assess arterial function and stiffness, such as flow-

For peer review only - http://bmjopen.bmj.com/site/about/guidelines.xhtml

mediated dilation and pulse-wave velocity, are not currently feasible in the very young due to limited compliance and technical inconveniences.[18]

Implications and future research

Our results suggest that intrauterine exposure to GDM does not induce changes in the carotid artery structure that are detectable with conventional ultrasound techniques at birth and may not be linked to early vascular aging at this arterial site in the short term. Measurements at other arterial sites, such as the aorta,[32] may be more useful to investigate early or subtle abnormalities related to accelerated vascular aging or subclinical atherosclerosis. A long-term follow-up that includes complementary vascular measures, for instance, endothelium-dependent and endothelium-independent vasodilation or large-artery stiffness,[20] may shed further light on the cardiovascular health of children born to mothers with GDM.

Patient and public involvement

There was no patient or public involvement in the design, conduct, analysis, or reporting of this study's findings.

Author affiliations

[1]Population Health Laboratory (#PopHealthLab), University of Fribourg, Fribourg, Switzerland;

[2]Department of Epidemiology and Health Services, Center for Primary Care and Public Health (UNISANTÉ), University of Lausanne, Lausanne, Switzerland;

[3]Paediatric Cardiology Unit, Woman-Mother-Child Department, Lausanne University Hospital (CHUV), Lausanne, Switzerland;

For peer review only

[4]Institute of Primary Health Care (BIHAM), University of Bern, Bern, Switzerland;
[5]School of Global and Population Health, McGill University, Montréal, Canada

Authors' contributions

AC, NS, SDB, and YM designed the study and the data collection procedures with input from SEY, AME. SEY and AME collected baseline characteristics for participants without GDM. SDB and NS collected neonatal cardiovascular characteristics for all participants. SEY performed data management and curation. AME carried out the statistical analyses with input and supervision from AC. AME wrote the first draft of the manuscript with input from AC and NS. SDB, SEY, YM made critical revisions to the manuscript for important intellectual content. All authors read and approved the content of the manuscript.

Acknowledgements

The study teams of *MySweetHeart* Cohort and *MySweetHeart* Trial form *MySweetHeart* Research group and collaborated on data collection, management, and curation. We thank the following members of *MySweetHeart* Research Group, listed in alphabetical order: Amar Arhab, Pascal Bovet, Arnaud Chiolero, Stefano Di Bernardo, Adina Mihaela Epure, Sandrine Estoppey Younes, Leah Gilbert, Justine Gross, Antje Horsch, Stefano Lanzi, Seyda Mayerat, Yvan Mivelaz, Jardena J. Puder, Dan Quansah, Jean-Benoit Rossel, Nicole Sekarski, Umberto Simeoni, Bobby Stuijfzand, Yvan Vial.

Funding

For peer review only

This study was funded by the Swiss National Science Foundation (www.snf.ch; *MySweetHeart* Cohort project number 32003B-163240; *MySweetHeart* Trial project number 32003B_176119). The funder had no role in the study design, data collection and analysis, or interpretation of results.

Competing interests

None declared.

Consent for publication

Not applicable.

Ethics approval

Ethical approval was obtained through the Ethics Committee for Human Research of the Canton of Vaud (2016–00745).

Data availability statement

Data could be made available by the principal investigator and corresponding author (Prof Nicole Sekarski: nicole.sekarski@chuv.ch) on reasonable request.

For peer review only

For peer review only - http://bmjopen.bmj.com/site/about/guidelines.xhtml

REFERENCES

1. Reece EA, Leguizamon G, Wiznitzer A. Gestational diabetes: the need for a common ground. *Lancet* 2009;373(9677):1789-97. doi: 10.1016/s0140-6736(09)60515-8.
2. Reece EA. The fetal and maternal consequences of gestational diabetes mellitus. *J Matern Fetal Neonatal Med* 2010;23(3):199-203. doi: 10.3109/14767050903550659.
3. American Diabetes Association (ADA). 2. Classification and Diagnosis of Diabetes: Standards of Medical Care in Diabetes—2021. *Diabetes Care* 2021;44(Supplement 1):S15-S33. doi: 10.2337/dc21-S002.
4. International Diabetes Federation. IDF Diabetes Atlas. 9th edition. Brussels, Belgium: International Diabetes Federation, 2019.
5. Damm P, Houshmand-Oeregaard A, Kelstrup L, et al. Gestational diabetes mellitus and long-term consequences for mother and offspring: a view from Denmark. *Diabetologia* 2016;59(7):1396-99. doi: 10.1007/s00125-016-3985-5.
6. Li JW, He SY, Liu P, et al. Association of gestational diabetes mellitus (GDM) with subclinical atherosclerosis: a systemic review and meta-analysis. *BMC Cardiovasc Disord* 2014;14:132. doi: 10.1186/1471-2261-14-132.
7. Tobias DK, Stuart JJ, Li S, et al. Association of History of Gestational Diabetes With Long-term Cardiovascular Disease Risk in a Large Prospective Cohort of US Women. *JAMA Intern Med* 2017;177(12):1735-42. doi: 10.1001/jamainternmed.2017.2790.
8. Epure AM, Rios-Leyvraz M, Anker D, et al. Risk factors during first 1,000 days of life for carotid intima-media thickness in infants, children, and adolescents: A

For peer review only

For peer review only - http://bmjopen.bmj.com/site/about/guidelines.xhtml

systematic review with meta-analyses. *PLoS Med* 2020;17(11):e1003414. doi: 10.1371/journal.pmed.1003414.

9. Giannopoulou EZ, Doundoulakis I, Antza C, et al. Subclinical arterial damage in children and adolescents with type 1 diabetes: A systematic review and meta-analysis. *Pediatr Diabetes* 2019;20(6):668-77. doi: 10.1111/pedi.12874.

10. Hanson MA, Gluckman PD. Early developmental conditioning of later health and disease: physiology or pathophysiology? *Physiol Rev* 2014;94(4):1027-76. doi: 10.1152/physrev.00029.2013.

11. Aceti A, Santhakumaran S, Logan KM, et al. The diabetic pregnancy and offspring blood pressure in childhood: a systematic review and meta-analysis. *Diabetologia* 2012;55(11):3114-27. doi: 10.1007/s00125-012-2689-8.

12. Mivelaz Y, Di Bernardo S, Boulos Ksontini T, et al. Feasibility and reliability of carotid intima-media thickness measurements in nonsedated infants. *J Hypertens* 2016;34(11):2227-32. doi: 10.1097/hjh.0000000000001065.

13. Di Bernardo S, Mivelaz Y, Epure AM, et al. Assessing the consequences of gestational diabetes mellitus on offspring's cardiovascular health: MySweetHeart Cohort study protocol, Switzerland. *BMJ Open* 2017;7(11):e016972. doi: 10.1136/bmjopen-2017-016972.

14. Horsch A, Gilbert L, Lanzi S, et al. Improving cardiometabolic and mental health in women with gestational diabetes mellitus and their offspring: study protocol for MySweetHeart Trial, a randomised controlled trial. *BMJ Open* 2018;8(2):e020462. doi: 10.1136/bmjopen-2017-020462.

15. Metzger BE, Gabbe SG, Persson B, et al. International association of diabetes and pregnancy study groups recommendations on the diagnosis and classification

For peer review only

For peer review only - http://bmjopen.bmj.com/site/about/guidelines.xhtml

of hyperglycemia in pregnancy. *Diabetes Care* 2010;33(3):676-82. doi: 10.2337/dc09-1848.

16. Urbina EM, Williams RV, Alpert BS, et al. Noninvasive assessment of subclinical atherosclerosis in children and adolescents: recommendations for standard assessment for clinical research: a scientific statement from the American Heart Association. *Hypertension* 2009;54(5):919-50. doi: 10.1161/hypertensionaha.109.192639.
17. Mosteller RD. Simplified calculation of body-surface area. *N Engl J Med* 1987;317(17):1098. doi: 10.1056/nejm198710223171717.
18. Torigoe T, Dallaire F, Slorach C, et al. New Comprehensive Reference Values for Arterial Vascular Parameters in Children. *J Am Soc Echocardiogr* 2020 doi: 10.1016/j.echo.2020.03.001.
19. Sarkola T, Manlhiot C, Slorach C, et al. Evolution of the arterial structure and function from infancy to adolescence is related to anthropometric and blood pressure changes. *Circulation* 2012;126(21).
20. Sundholm JKM, Litwin L, Rönö K, et al. Maternal obesity and gestational diabetes: Impact on arterial wall layer thickness and stiffness in early childhood - RADIEL study six-year follow-up. *Atherosclerosis* 2019 doi: 10.1016/j.atherosclerosis.2019.01.037.
21. Ayer JG, Harmer JA, Nakhla S, et al. HDL-cholesterol, blood pressure, and asymmetric dimethylarginine are significantly associated with arterial wall thickness in children. *Arterioscler Thromb Vasc Biol* 2009;29(6):943-49. doi: 10.1161/ATVBAHA.109.184184.

For peer review only

22. Atabek ME, Çağan HH, Eklioğlu BS, et al. Absence of increase in carotid artery Intima-Media thickness in infants of diabetic mothers. *J Clin Res Pediatr Endocrinol* 2011;3(3):144-48. doi: 10.4274/jcrpe.v3i3.28.

23. World Health Organization. Diagnostic criteria and classification of hyperglycaemia first detected in pregnancy: a World Health Organization Guideline. *Diabetes Res Clin Pract* 2014;103(3):341-63. doi: 10.1016/j.diabres.2013.10.012.

24. Yuen L, Saeedi P, Riaz M, et al. Projections of the prevalence of hyperglycaemia in pregnancy in 2019 and beyond: Results from the International Diabetes Federation Diabetes Atlas, 9th edition. *Diabetes Res Clin Pract* 2019;157:107841. doi: 10.1016/j.diabres.2019.107841.

25. Dalla Pozza R, Ehringer-Schetitska D, Fritsch P, et al. Intima media thickness measurement in children: A statement from the Association for European Paediatric Cardiology (AEPC) Working Group on Cardiovascular Prevention endorsed by the Association for European Paediatric Cardiology. *Atherosclerosis* 2015;238(2):380-7. doi: 10.1016/j.atherosclerosis.2014.12.029.

26. Agarwal MM, Dhatt GS, Shah SM. Gestational Diabetes Mellitus: Simplifying the International Association of Diabetes and Pregnancy diagnostic algorithm using fasting plasma glucose. *Diabetes Care* 2010;33(9):2018. doi: 10.2337/dc10-0572.

27. Mahdavian M, Hivert M-F, Baillargeon J-P, et al. Gestational Diabetes Mellitus: Simplifying the International Association of Diabetes and Pregnancy Diagnostic Algorithm Using Fasting Plasma Glucose. Comment on Agarwal, Dhatt, and Shah. *Diabetes Care* 2010;33(11):e145. doi: 10.2337/dc10-1454.

For peer review only

28. McIntyre HD, Jensen DM, Jensen RC, et al. Gestational Diabetes Mellitus: Does One Size Fit All? A Challenge to Uniform Worldwide Diagnostic Thresholds. *Diabetes Care* 2018;41(7):1339-42. doi: 10.2337/dc17-2393.

29. Brodersen J, Schwartz LM, Heneghan C, et al. Overdiagnosis: what it is and what it isn't. *BMJ Evidence-Based Medicine* 2018;23(1):1. doi: 10.1136/ebmed-2017-110886.

30. Olander RFW, Sundholm JKM, Ojala TH, et al. Neonatal Arterial Morphology Is Related to Body Size in Abnormal Human Fetal Growth. *Circ Cardiovasc Imaging* 2016;9(9) doi: 10.1161/CIRCIMAGING.116.004657.

31. Sarkola T, Slorach C, Hui W, et al. Transcutaneous very-high resolution ultrasound for the quantification of carotid arterial intima-media thickness in children - feasibility and comparison with conventional high resolution vascular ultrasound imaging. *Atherosclerosis* 2012;224(1):102-7. doi: 10.1016/j.atherosclerosis.2012.06.054.

32. Koklu E, Akcakus M, Kurtoglu S, et al. Aortic intima-media thickness and lipid profile in macrosomic newborns. *Eur J Pediatr* 2007;166(4):333-8. doi: 10.1007/s00431-006-0243-8.

For peer review only - http://bmjopen.bmj.com/site/about/guidelines.xhtml

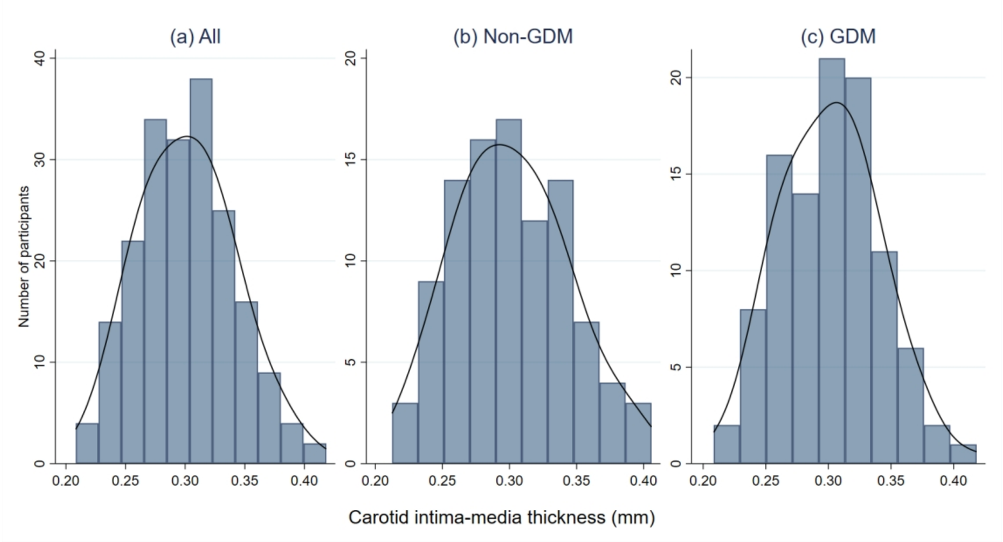

This figure shows the distribution of CIMT values in our sample, overall (n=200) and by GDM exposure (Non-GDM: n=99; GDM: n=101). The black line represents the kernel density estimate. Abbreviations: CIMT, carotid intima-media thickness; GDM, gestational diabetes mellitus.

599x326mm (72 x 72 DPI)

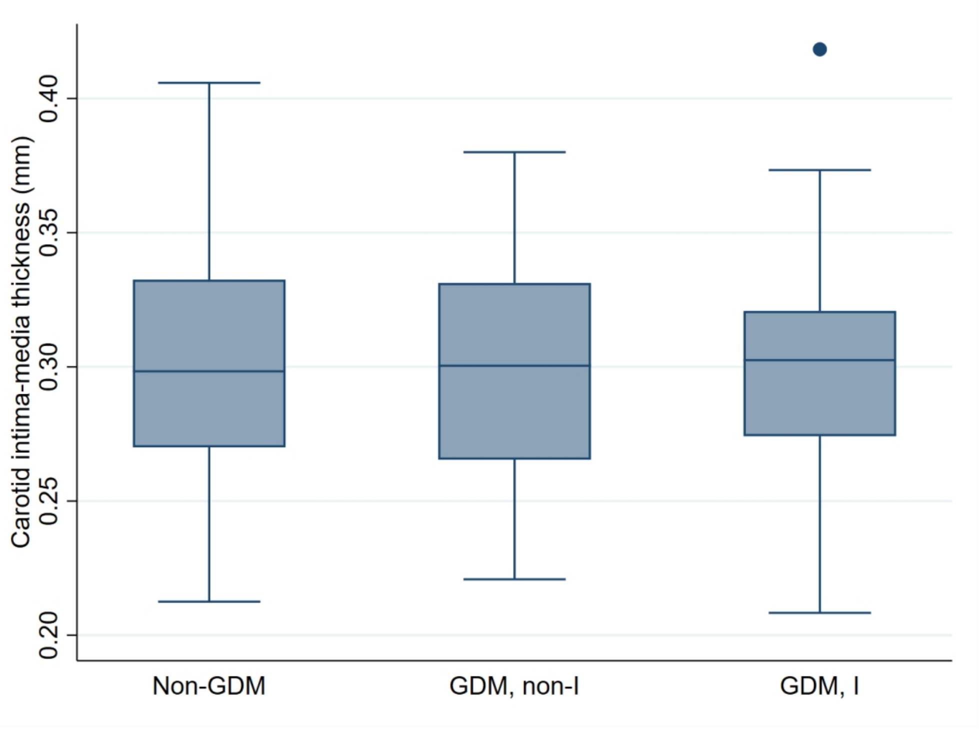

This figure shows the distribution of CIMT in the offspring of women without GDM (Non-GDM; n=99) and the offspring of women with GDM who were assigned to no intervention (GDM, non-I; n=48) or to a lifestyle and psychosocial intervention (GDM, I; n=53) as part of their participation in the MySweetHeart Trial. Abbreviations: CIMT, carotid intima-media thickness; GDM, gestational diabetes mellitus; I, intervention.

543x395mm (72 x 72 DPI)

SUPPLEMENTARY MATERIAL

Gestational diabetes mellitus and offspring's carotid intima-media thickness at birth: *MySweetHeart* Cohort Study

Adina Mihaela Epure[1,2], Stefano Di Bernardo[3], Yvan Mivelaz[3], Sandrine Estoppey Younes[2], Arnaud Chiolero[1,4,5¶], Nicole Sekarski[3¶], on behalf of *MySweetHeart* Research Group

[1]Population Health Laboratory (#PopHealthLab), University of Fribourg, Fribourg, Switzerland;

[2]Department of Epidemiology and Health Services, Center for Primary Care and Public Health (UNISANTÉ), University of Lausanne, Lausanne, Switzerland;

[3]Paediatric Cardiology Unit, Woman-Mother-Child Department, Lausanne University Hospital (CHUV), Lausanne, Switzerland;

[4]Institute of Primary Health Care (BIHAM), University of Bern, Bern, Switzerland;

[5]School of Global and Population Health, McGill University, Montréal, Canada

¶ These authors contributed equally to this work.

For peer review only - http://bmjopen.bmj.com/site/about/guidelines.xhtml

For peer review only

Table S1 Differences in CIMT at birth by GDM exposure and assignment to a lifestyle and psychosocial intervention.

	Mean (sd), mm	**Model 1 (n=200)**		**Model 2 (n=165)**		**Model 3 (n=165)**	
		Difference (95% CI), mm	p	Difference (95% CI), mm	p	Difference (95% CI), mm	p
Non-GDM	0.30 (0.04)	ref	ref	ref	ref	ref	ref
GDM, non-I	0.30 (0.04)	0.00 (-0.01 to 0.01)	0.94	0.00 (-0.02 to 0.02)	0.93	0.00 (-0.02 to 0.01)	0.91
GDM, I	0.30 (0.04)	0.00 (-0.01 to 0.01)	0.98	-0.01 (-0.03 to 0.01)	0.19	-0.01 (-0.02 to 0.01)	0.25

Model 1: unadjusted estimates.

Model 2: estimates adjusted for maternal pre-pregnancy BMI, education, and tobacco smoking; offspring family history of diabetes and sex.

Model 3: estimates adjusted for maternal pre-pregnancy BMI, education, and tobacco smoking; offspring family history of diabetes, sex, body surface area and age at CIMT assessment.

Abbreviations: CI, confidence interval; CIMT, carotid intima-media thickness; GDM, gestational diabetes mellitus; I, intervention; n, total number of participants; p, p-value; sd, standard deviation; ref, reference group.

Note: Similar results were obtained when Model 1 was run in the sample (n=165) with data on outcome, exposure, and all covariates included in Models 2 and 3 (GDM, non-I: 0.00 mm (95% CI: -0.01 to 0.01; p=0.99); GDM, I: -0.01 mm (95% CI: -0.02 to 0.01; p=0.29)).

For peer review only

STROBE 2007 (v4) Statement—Checklist of items that should be included in reports of *cohort studies* [1]

Section/Topic	Item #	Recommendation	Reported in section [page # in Main text]
**Title and abstract**	1	(*a*) Indicate the study's design with a commonly used term in the title or the abstract	Title page [page 1]
		(*b*) Provide in the abstract an informative and balanced summary of what was done and what was found	Abstract [page 2-3]
**Introduction**			
Background/rationale	2	Explain the scientific background and rationale for the investigation being reported	Introduction (paragraphs 1-2) [page 5]
Objectives	3	State specific objectives, including any prespecified hypotheses	Introduction (paragraph 2) [page 5]
**Methods**			
Study design	4	Present key elements of study design early in the paper	- Methods (subheadings: Study design and setting; Study population) [page 6]<br>- Published protocol (see reference [2])
Setting	5	Describe the setting, locations, and relevant dates, including periods of recruitment, exposure, follow-up, and data collection	- Methods (subheadings: Study design and setting; Study population; Data collection) [page 6-9]<br>- Results (subheadings: Characteristics of study participants) [page 9]<br>- Published protocol (see reference [2])
Participants	6	(*a*) Give the eligibility criteria, and the sources and methods of selection of participants. Describe methods of follow-up	- Methods (subheadings: Study population) [page 6]<br>- Published protocol (see reference [2])
		(*b*) For matched studies, give matching criteria and number of exposed and unexposed	- N/A
Variables	7	Clearly define all outcomes, exposures, predictors, potential confounders, and effect modifiers. Give diagnostic criteria, if applicable	- Methods (subheadings: Data collection; Data analysis) [page 6-9]
Data sources/ measurement	8*	For each variable of interest, give sources of data and details of methods of assessment (measurement). Describe comparability of assessment methods if there is more than one group	- Methods (subheadings: Data collection) [page 6-8]
Bias	9	Describe any efforts to address potential sources of bias	- Methods (subheadings: Data analysis) [page 9]<br>- Discussion (subheadings: Strengths and limitations) [page 15-16]

For peer review only

Study size	10	Explain how the study size was arrived at	- Published protocol[2]
Quantitative variables	11	Explain how quantitative variables were handled in the analyses. If applicable, describe which groupings were chosen and why	- Methods (subheadings: Data analysis) [page 9]
Statistical methods	12	(*a*) Describe all statistical methods, including those used to control for confounding	- Methods (subheadings: Data analysis) [page 9]
		(*b*) Describe any methods used to examine subgroups and interactions	
		(*c*) Explain how missing data were addressed	- Methods (subheadings: Study population; Data analysis) [page 6; 9]<br>- Table 1 footnote [page 11]
		(*d*) If applicable, explain how loss to follow-up was addressed	- Methods (subheadings: Study population) [page 6]
		(*e*) Describe any sensitivity analyses	- Supplementary material (Table S1)
**Results**			
Participants	13*	(a) Report numbers of individuals at each stage of study—eg numbers potentially eligible, examined for eligibility, confirmed eligible, included in the study, completing follow-up, and analysed	- Results (subheadings: Characteristics of study participants) [page 9-10]
		(b) Give reasons for non-participation at each stage	
		(c) Consider use of a flow diagram	
Descriptive data	14*	(a) Give characteristics of study participants (eg demographic, clinical, social) and information on exposures and potential confounders	- Results (subheadings: Characteristics of study participants; Table 1) [page 10-11]
		(b) Indicate number of participants with missing data for each variable of interest	- Results (Table 1) [page 11]
		(c) Summarise follow-up time (eg, average and total amount)	- Methods (subheadings: Study population; Carotid ultrasound and CIMT measurement) [page 6-7]
Outcome data	15*	Report numbers of outcome events or summary measures over time	- Results (Table 2) [page 13]
Main results	16	(*a*) Give unadjusted estimates and, if applicable, confounder-adjusted estimates and their precision (eg, 95% confidence interval). Make clear which confounders were adjusted for and why they were included	- Results (Table 2) [page 13]
		(*b*) Report category boundaries when continuous variables were categorized	- Results (Table 1) [page 11]
		(*c*) If relevant, consider translating estimates of relative risk into absolute risk for a meaningful time period	- N/A
Other analyses	17	Report other analyses done—eg analyses of subgroups and interactions, and sensitivity analyses	- Supplementary material (Table S1)

For peer review only

**Discussion**			
Key results	18	Summarise key results with reference to study objectives	- Discussion (Summary of findings and comparison with other studies) [page 14]
**Limitations**			
Interpretation	20	Give a cautious overall interpretation of results considering objectives, limitations, multiplicity of analyses, results from similar studies, and other relevant evidence	- Discussion (Summary of findings and comparison with other studies; Strengths and limitations) [page 14-16]
Generalisability	21	Discuss the generalisability (external validity) of the study results	- Discussion (Strengths and limitations) [page 15-16]
**Other information**			
Funding	22	Give the source of funding and the role of the funders for the present study and, if applicable, for the original study on which the present article is based	- Funding statement [page 19]

*Give information separately for cases and controls in case-control studies and, if applicable, for exposed and unexposed groups in cohort and cross-sectional studies.

Note: Information on the STROBE Initiative is available at www.strobe-statement.org.

References

1. von Elm E, Altman DG, Egger M, et al. The Strengthening the Reporting of Observational Studies in Epidemiology (STROBE) Statement: Guidelines for Reporting Observational Studies. *PLOS Medicine* 2007;4(10):e296. doi: 10.1371/journal.pmed.0040296.
2. Di Bernardo S, Mivelaz Y, Epure AM, et al. Assessing the consequences of gestational diabetes mellitus on offspring's cardiovascular health: MySweetHeart Cohort study protocol, Switzerland. *BMJ Open* 2017;7(11):e016972. doi: 10.1136/bmjopen-2017-016972.

For peer review only

BMJ Open

Gestational diabetes mellitus and offspring's carotid intima-media thickness at birth: MySweetHeart Cohort Study

Journal:	*BMJ Open*
Manuscript ID	bmjopen-2022-061649.R1
Article Type:	Original research
Date Submitted by the Author:	17-Jun-2022
Complete List of Authors:	Epure, Adina Mihaela; University of Fribourg, Population Health Laboratory; University of Lausanne, Center for Primary Care and Public Health, Department of Epidemiology and Health Services<br>Di Bernardo, Stefano; Lausanne University Hospital, Paediatric Cardiology Unit, Department Woman-Mother-Child<br>Mivelaz, Yvan; Lausanne University Hospital, Paediatric Cardiology Unit, Department Woman-Mother-Child<br>Estoppey Younes, Sandrine; University of Lausanne, Center for Primary Care and Public Health, Department of Epidemiology and Health Services<br>Chiolero, Arnaud; University of Fribourg, Population Health Laboratory; University of Bern, Institute of Primary Health Care<br>Sekarski, Nicole; Lausanne University Hospital, Paediatric Cardiology Unit, Woman-Mother-Child Department
**Primary Subject Heading**:	Cardiovascular medicine
Secondary Subject Heading:	Epidemiology, Paediatrics
Keywords:	Cardiovascular imaging < RADIOLOGY & IMAGING, Paediatric cardiology < CARDIOLOGY, Diabetes in pregnancy < DIABETES & ENDOCRINOLOGY, NEONATOLOGY, Cardiac Epidemiology < CARDIOLOGY

SCHOLARONE™
Manuscripts

I, the Submitting Author has the right to grant and does grant on behalf of all authors of the Work (as defined in the below author licence), an exclusive licence and/or a non-exclusive licence for contributions from authors who are: i) UK Crown employees; ii) where BMJ has agreed a CC-BY licence shall apply, and/or iii) in accordance with the terms applicable for US Federal Government officers or employees acting as part of their official duties; on a worldwide, perpetual, irrevocable, royalty-free basis to BMJ Publishing Group Ltd ("BMJ") its licensees and where the relevant Journal is co-owned by BMJ to the co-owners of the Journal, to publish the Work in this journal and any other BMJ products and to exploit all rights, as set out in our licence.

The Submitting Author accepts and understands that any supply made under these terms is made by BMJ to the Submitting Author unless you are acting as an employee on behalf of your employer or a postgraduate student of an affiliated institution which is paying any applicable article publishing charge ("APC") for Open Access articles. Where the Submitting Author wishes to make the Work available on an Open Access basis (and intends to pay the relevant APC), the terms of reuse of such Open Access shall be governed by a Creative Commons licence – details of these licences and which Creative Commons licence will apply to this Work are set out in our licence referred to above.

Other than as permitted in any relevant BMJ Author's Self Archiving Policies, I confirm this Work has not been accepted for publication elsewhere, is not being considered for publication elsewhere and does not duplicate material already published. I confirm all authors consent to publication of this Work and authorise the granting of this licence.

For peer review only

Gestational diabetes mellitus and offspring's carotid intima-media thickness at birth: *MySweetHeart* Cohort Study

Adina Mihaela Epure[1,2], Stefano Di Bernardo[3], Yvan Mivelaz[3], Sandrine Estoppey Younes[2], Arnaud Chiolero[1,4,5¶], Nicole Sekarski[3¶*], on behalf of *MySweetHeart* Research Group

[1]Population Health Laboratory (#PopHealthLab), University of Fribourg, Fribourg, Switzerland;

[2]Department of Epidemiology and Health Services, Center for Primary Care and Public Health (UNISANTÉ), University of Lausanne, Lausanne, Switzerland;

[3]Paediatric Cardiology Unit, Woman-Mother-Child Department, Lausanne University Hospital (CHUV), Lausanne, Switzerland;

[4]Institute of Primary Health Care (BIHAM), University of Bern, Bern, Switzerland;

[5]School of Global and Population Health, McGill University, Montréal, Canada

¶ These authors contributed equally to this work.

* **Corresponding author:** Prof Nicole Sekarski, nicole.sekarski@chuv.ch, Lausanne University Hospital (CHUV), 46 Route du Bugnon, Lausanne, 1011, Switzerland

Word count (excluding title page, abstract and article summary, references, figure legends and tables): 2'924

For peer review only

ABSTRACT

Objective Hyperglycemia during pregnancy is associated with cardiometabolic risks for the mother and the offspring. Mothers with gestational diabetes mellitus (GDM) have signs of subclinical atherosclerosis, including increased carotid intima-media thickness (CIMT). We assessed whether GDM is associated with increased CIMT in the offspring at birth.

Design and setting *MySweetHeart* Cohort is a prospective cohort study conducted in Switzerland.

Participants, exposure and outcome measures This work included pregnant women with and without GDM at 24 to 32 weeks of gestation and their singleton live-born offspring with data on the primary outcome of CIMT. GDM was diagnosed based on the criteria of the International Association of Diabetes and Pregnancy Study Groups. Offspring's CIMT was measured by ultrasonography after birth (range: 1 to 19 days).

Results Data on CIMT were available for 99 offspring of women without GDM and 101 offspring of women with GDM. Maternal age ranged from 18 to 47 years. Some 16% of women with GDM and 6% of women without GDM were obese. Smoking during pregnancy was more frequent among women with GDM (18%) than among those without GDM (4%). Neonatal characteristics were comparable between the 2 groups. The difference in CIMT between offspring of women with and without GDM was of 0.00 mm (95% CI: -0.01 to 0.01; p=0.96) and remained similar upon adjustment for potential confounding factors, such as maternal pre-pregnancy BMI, maternal education,

For peer review only

smoking during pregnancy, family history of diabetes, as well as offspring's sex, age, and body surface area (0.00 mm (95% CI: -0.02 to 0.01; p=0.45)).

Conclusions We found no evidence of increased CIMT in neonates exposed to GDM. A longer-term follow-up that includes additional vascular measures, such as endothelial function or arterial stiffness, may shed further light on the cardiovascular health trajectories in children born to mothers with GDM.

Registration ClinicalTrials.gov (NCT02872974)

Keywords gestational diabetes; carotid intima-media thickness; cardiovascular prevention; child; neonate

List of abbreviations BMI, body mass index; CIMT, carotid intima-media thickness; CVD, cardiovascular disease; CHUV, Lausanne University Hospital; DOHaD, developmental origins of health and disease; FPG, fasting plasma glucose; GDM, gestational diabetes mellitus; HbA1c, glycated hemoglobin; IADPSG, International Association of Diabetes and Pregnancy Study Groups; I, intervention; OGTT, oral glucose tolerance test.

For peer review only

Strengths and limitations of this study

- One important strength of this study is represented by its prospective design and the enrollment of participants at the time of gestational diabetes diagnosis.
- Carotid intima-media thickness was measured in non-sedated neonates by experienced pediatric cardiologists using automated methods with manual tracing adjustment, in accordance with published guidelines.
- Limitations of this study include the relatively small sample size, the possibility of residual confounding and the limited generalizability.

For peer review only

INTRODUCTION

Gestational diabetes mellitus (GDM) is a state of hyperglycemia with onset or first recognition during pregnancy.[1-3] The prevalence of hyperglycemia during pregnancy has increased in recent decades, being estimated at 16% worldwide in 2019, with 84% of cases due to GDM.[4] GDM is associated with long-term metabolic consequences for both the mother and the offspring, such as type 2 diabetes and obesity.[5] Women with GDM also have subclinical atherosclerosis and an increased risk for cardiovascular disease (CVD) later in life.[6,7] However, little is known about the cardiovascular risk of their offspring.

CIMT is a surrogate marker of atherosclerosis, which has been shown to be increased in children exposed to risk factors in the first 1000 days of life, such as poor fetal growth,[8] as well as in children with type 1 diabetes.[9] From a developmental origins of health and disease (DOHaD) perspective,[10] exposure to adverse experiences in early life may produce lifelong adaptations in the organs' structure and function and may program the risk for CVD. For instance, a systematic review and meta-analysis showed that GDM was associated with a higher systolic blood pressure in childhood.[11] Whether GDM has an impact on children's CIMT is not clearly established. The evidence is scarce notably in the very young children although CIMT measurement is feasible from birth and could help discern between changes that occur before or after birth.[12] To fill this gap, we conducted *MySweetHeart Cohort* study to assess the early life cardiovascular consequences of GDM.[13] Herein, we evaluated CIMT at birth in offspring of mothers with and without GDM.

For peer review only - http://bmjopen.bmj.com/site/about/guidelines.xhtml

METHODS

Study design and setting

MySweetHeart Cohort is a prospective cohort study conducted at the Lausanne University Hospital (CHUV), Switzerland. The study has been registered with ClinicalTrials.gov (clinicaltrials.gov/ct2/show/NCT02872974) and the study protocol has been published.[13] Ethical approval was granted by the Ethics Committee for Human Research of the Canton de Vaud (study number 2016-00745).

Study population

This cohort included pregnant women between 24 and 32 weeks of gestation, with and without GDM. Other inclusion criteria were age 18 years or more and understanding French or English. The exclusion criteria were pre-existing diabetes mellitus, strict bed rest, or severe mental disorders. To facilitate recruitment and share resources, a collaboration was established with *MySweetHeart Trial*,[14] a randomized controlled trial assessing the effect of a lifestyle and psychosocial intervention on cardiometabolic outcomes of women with GDM and their offspring. As such, women with GDM were invited to contribute to both studies. Participating women with and without GDM were included in the current analysis if CIMT data for their live-born singleton neonates were available. All families gave a signed informed consent for use of their data.

Data collection

GDM screening

Pregnant women screened at the prenatal care clinic of the CHUV had a fasting plasma glucose (FPG) test between 24 and 28 weeks of gestation and GDM was diagnosed if the test result was ≥ 5.1 mmol/L.[13] If FPG was < 5.1 mmol/L, but ≥ 4.4 mmol/L,

For peer review only

women had a 2-hour 75 g oral glucose tolerance test (OGTT) and GDM was diagnosed based on the criteria of the International Association of Diabetes and Pregnancy Study Groups (IADPSG).[15] Pregnant women screened by external obstetricians in the Canton of Vaud underwent the same procedure or directly a 2-hour 75 g OGTT.

Carotid ultrasound and CIMT measurement

A carotid ultrasound assessment was performed between 1 and 7 days of life in the majority of neonates (n=191). A small share (n=9) had the exam between 8 and 19 days of life due to organizational and logistical constraints. Parents were told to feed and burp their offspring ahead of the carotid ultrasound to make them more relaxed. Feeding or administration of a 30% glucose solution were used to comfort the neonates if they became agitated during the exam. The exam took place in a dark and quiet room and a cloth was placed under the neonates' shoulders to facilitate the extension of the neck.

Ultrasound image acquisition and analysis were performed by 2 experienced pediatric cardiologists who were blinded to the maternal glycemic status. Images were acquired in B-mode with no harmonics, sonoCT, dynamic range of 60dB, at a frame rate of 100-120Hz, with a depth of 1-2 cm. The right and left carotid arteries were scanned using a Philips EPIC echocardiograph (Philips Medical, Netherlands) with a L 15-7 MHz high-resolution linear array transducer, according to the American Heart Association's recommendations for standard assessment of subclinical atherosclerosis in children and adolescents.[16] Each observer recorded three consecutive 3-second cine loops from 2 different angles on each side, which were stored as native DICOM for subsequent offline analyses (QLab, Philips Medical, Netherlands). Whenever image

For peer review only - http://bmjopen.bmj.com/site/about/guidelines.xhtml

quality was optimal enough, 6 right and 6 left frames were selected and, for each, the maximal IMT of the common carotid artery far wall was measured. Measurements were performed over a 1-cm region of interest proximal to the carotid bulb, on or closest to the R-wave of the electrocardiogram, using a semi-automated edge detection software with manual tracing adjustment when needed. The mean of 12 maximal CIMT measurements was used in the analysis for the majority of neonates (n=170). Two neonates had only one measurement available, whereas the rest had between 2 and 11 measurements that were averaged. A good interobserver reliability (coefficient of variation=5.9%) for measurements in non-sedated infants was proven in our laboratory previously.[12]

Other sample characteristics

Data on maternal characteristics (age, country of origin, education, smoking during pregnancy, pre-pregnancy weight and height, or parity) and family history of diabetes were record-based or self-reported by the mother at a researcher-administered interview upon inclusion in the study. Smoking during pregnancy was defined as a mother who was an active tobacco smoker at study baseline, i.e., between 24 and 32 weeks of gestation. A maternal blood sampling was also performed at baseline and glycated hemoglobin (HbA1c) was measured. Pre-pregnancy body mass index (BMI) was computed by dividing the pre-pregnancy weight (kg) by the squared height (m^2). Delivery data such as newborn sex, anthropometry, gestational age, or mode of delivery were obtained from the medical records. Neonatal weight, length and blood pressure were measured by the study team at the time of the carotid ultrasound. Body surface area (m^2) was computed using the Mosteller equation.[17] One systolic and diastolic blood pressure measurement was taken from the right upper arm, in a supine

For peer review only - http://bmjopen.bmj.com/site/about/guidelines.xhtml

position, using a clinically validated and regularly calibrated oscillometric sphygmomanometer (Accutorr Plus; Datascope, Paramus, New Jersey, USA) with neonate cuffs.

Data analysis

Descriptive statistics on study participants are reported as percentages (%) or as mean, standard deviation, minimum and maximum values. The relationship of GDM with CIMT was evaluated by a set of linear regression models with and without adjustment for potential confounders, i.e., baseline covariates associated with metabolic and cardiovascular risks, offspring's sex, and anthropometry at CIMT assessment. Potential confounders were maternal pre-pregnancy BMI, maternal education (university/no university), smoking during pregnancy (yes/no), and family history of diabetes (yes/no). The variable family history of diabetes summarized disease occurrence in a 1st degree relative of the mother, 1st degree relative of the father or in the father himself and assumed missing data in any of these variables as no history of diabetes unless values for all 3 variables were missing. To account for differences in body size,[18,19] we adjusted for body surface area and age at CIMT assessment. All statistical analyses were performed in Stata 16 (Stata Corporation, Texas, USA).

RESULTS

Characteristics of study participants

Data collection started in September 2016 and ended in October 2020. A total of 137 participants without GDM exposure and 212 participants with GDM exposure were recruited in the study. Some 101 neonates without GDM exposure and 117 neonates

For peer review only - http://bmjopen.bmj.com/site/about/guidelines.xhtml

with GDM exposure attended the cardiovascular follow-up visit early after birth. Of these, 200 singleton neonates born at more than 36 weeks of gestation (non-GDM: n=99; GDM: n=101) had CIMT measurements and constitute the analytic sample for the current analysis.

Family and neonatal characteristics of study participants are presented in Table 1. The maternal characteristics were generally comparable between the non-GDM and GDM groups. The majority of women were non-Swiss and their age ranged from 18 to 47 years. Approximately half of the women in each group had a high level of education and no previous deliveries. More women with GDM (16%) were obese (pre-pregnancy BMI≥ 30 kg/m^2) compared to women without GDM (6%). Smoking during pregnancy was more frequent among women with GDM (18%) than among those without GDM (4%). Offspring of women with and without GDM had similar neonatal characteristics, such as sex, gestational age, birth weight, length, or blood pressure. The majority were born at term, i.e., between 37 and 41 weeks (GDM: 96%; non-GDM: 98%) and a small share had macrosomia, i.e., a birth weight higher than 4’000 g (GDM: 6%; non-GDM: 5%). Offspring of women with GDM (46%) had a higher frequency of family history of diabetes compared to their non-GDM counterparts (24%).

For peer review only

Table 1 Characteristics of study participants by GDM exposure.

	Non-GDM[a] (n=99)				GDM[b] (n=101)			
	**mean or %**	**sd**	**min**	**max**	**mean or %**	**sd**	**min**	**max**
**MATERNAL**								
Age (years)	33	5	18	44	33	5	21	47
Swiss origin (%)	24				33			
University education (%)	60				55			
Primiparous (%)	55				48			
Smoking during pregnancy (%)	4				18			
Pre-pregnancy obesity (BMI ≥ 30 kg/m2) (%)	6				16			
HbA1C (%)	4.9	0.3	4.2	5.7	5.3	0.3	4.7	7.2
**NEONATAL**								
Male (%)	52				53			
Cesarean section (%)	22				32			
Term birth (37 to 41 weeks) (%)	98				96			
Birth weight (g)	3'352	425	2'190	4'190	3'357	442	2'220	4'340
Macrosomia (Birth weight > 4'000 g) (%)	5				6			
Length (cm)	50	2	45	54	50	2	45	56
Body surface area (m^2)	0.21	0.02	0.16	0.25	0.21	0.02	0.17	0.26
Systolic BP (mmHg)	78	9	60	101	78	10	60	111
Diastolic BP (mmHg)	47	8	30	66	48	10	28	90
Family history of diabetes (%)	24				46			

Abbreviations: BMI, body mass index; BP, blood pressure; GDM, gestational diabetes mellitus; HbA1c, glycated hemoglobin n, total number of participants; max, maximum; min, minimum; sd, standard deviation.

[a] Non-GDM: Missing values for swiss origin (n=1), university education (n=2), pre-pregnancy obesity (n=1), HbA1c (n=13), cesarean section (n=4), term birth (n=10), systolic BP (n=1), diastolic BP (n=1); family history of diabetes (n=1).
[b] GDM: Missing values for age (n=3), swiss origin (n=3), university education (n=18), primiparous (n=3), smoking (n=5), pre-pregnancy obesity (n=4), HbA1c (n=5), male (n=16), cesarean section (n=6), term birth (n=16), birth weight (n=16); family history of diabetes (n=4).

For peer review only

GDM and CIMT at birth

The distribution of CIMT values is presented in Fig. 1, Fig. 2, and Fig. S2 in Supplementary Material. CIMT ranged from 0.21 to 0.42 mm, with a mean CIMT of 0.30 mm (sd 0.04) overall and in each of the studied groups (Table 2, Table S1 in Supplementary Material).

Fig. 1 Histograms of CIMT at birth, overall and by GDM exposure.

Figure legend This figure shows the distribution of CIMT values in our sample, overall (n=200) and by GDM exposure (Non-GDM: n=99; GDM: n=101). The black line represents the kernel density estimate. Abbreviations: CIMT, carotid intima-media thickness; GDM, gestational diabetes mellitus.

Fig. 2 Box plots of CIMT at birth by GDM exposure and assignment to a lifestyle and psychosocial intervention.

Figure legend This figure shows the distribution of CIMT in the offspring of women without GDM (*Non-GDM*; n=99) and the offspring of women with GDM who were assigned to no intervention (*GDM, non-I*; n=48) or to a lifestyle and psychosocial intervention (*GDM, I*; n=53) as part of their participation in the *MySweetHeart* Trial. The line inside the box represents the median value of the distribution, while the lower and upper boundaries of the box represent the first (Q1) and third quartiles (Q3), respectively. The interquartile range (IQR) corresponds to Q3 – Q1. The whiskers extend from either side of the box up to 1.5*IQR (i.e., Q1-1.5*IQR and Q3+1.5*IQR). Outliers are depicted as circles. Abbreviations: CIMT, carotid intima-media thickness; GDM, gestational diabetes mellitus; I, intervention.

For peer review only - http://bmjopen.bmj.com/site/about/guidelines.xhtml

245 **Table 2** The relationship of GDM with offspring's CIMT at birth.

	Mean (SD), mm	**Model 1 (n=200)**		**Model 2 (n=165)**		**Model 3 (n=165)**	
		Mean difference (95% CI), mm	p	Mean difference (95% CI), mm	p	Mean difference (95% CI), mm	p
Non-GDM	0.30 (0.04)	ref	ref	ref	ref	ref	ref
GDM	0.30 (0.04)	0.00 (-0.01 to 0.01)	0.96	0.00 (-0.02 to 0.01)	0.47	0.00 (-0.02 to 0.01)	0.45

Estimates were obtained from linear regression models with the following specification: Model 1: unadjusted estimates; Model 2: estimates adjusted for maternal pre-pregnancy BMI, education, and tobacco smoking; offspring family history of diabetes and sex; Model 3: estimates adjusted for maternal pre-pregnancy BMI, education, and tobacco smoking; offspring family history of diabetes, sex, body surface area and age at CIMT assessment. The outcome variable (i.e., CIMT) was continuous. The exposure variable was binary (GDM/Non-GDM; the reference category was Non-GDM). Similar results were obtained when Model 1 was run in the sample (n=165) with data on outcome, exposure, and all covariates included in Models 2 and 3 (GDM: 0.00 mm (95% CI: -0.02 to 0.01; p=0.54)). Abbreviations: CI, confidence interval; CIMT, carotid intima-media thickness; GDM, gestational diabetes mellitus; n, total number of participants; p, p-value; sd, standard deviation; ref, reference group.

For peer review only

The relationship of GDM with offspring's CIMT early after birth is presented in Table 2 and Fig. 3**.** In the unadjusted analysis (Model 1), the difference in CIMT between offspring of women with and without GDM was 0.00 mm (95% CI -0.01 to 0.01; p=0.96). Adjustment for offspring sex and potential confounding factors (Model 2), as well as for offspring's body surface area and age at CIMT assessment (Model 3), resulted in a difference of 0 mm (95% CI -0.02 to 0.01; p=0.45). When exposure to GDM was analyzed separately for offspring whose mothers were assigned or not to a lifestyle and psychosocial intervention as part of their participation in *MySweetHeart* Trial, results were similar to those presented above (Table S1 and Fig. S1 in Supplementary Material).

Fig 3 Illustration of the relationship of GDM with offspring's CIMT at birth through a forest plot.

Figure legend The boxes represent the mean differences in CIMT between offspring of women with and without GDM (i.e., GDM versus non-GDM). The horizontal lines represent the 95% CIs. The plot was constructed using regression estimates and models presented in Table 2. Model specification: Model 1 is unadjusted, while Models 2 and 3 are adjusted for various factors as described in the methods and footnote of Table 2. Abbreviations: CI, confidence interval; CIMT, carotid intima-media thickness; GDM, gestational diabetes mellitus.

DISCUSSION

Summary of findings and comparison with other studies

Our goal was to assess the relationship of GDM with neonatal CIMT. We found no evidence of an increased CIMT in neonates born to women with GDM as compared to

For peer review only

those born to women without GDM. Our findings are in line with other studies that evaluated CIMT after intrauterine exposure to maternal hyperglycemia. A recent meta-analysis pooled data from 3 studies and reported no clear evidence of increased CIMT in children exposed to maternal hyperglycemia compared to those not exposed (pooled standardized mean difference (SMD): 0.08 (95% CI -0.16 to 0.33)).[8] Two of these studies included 6-year and 8-year children, respectively, and found no difference in CIMT after exposure to GDM (SMD 0.00 (95% CI -0.28 to 0.28) at 6 years and 0.00 (95% CI -0.41 to 0.41) at 8 years).[8,20,21] The third study included neonates and found a slightly higher CIMT among those exposed to diabetes (SMD 0.46 (95% CI -0.07 to 1.00)),[8,22] but the imprecision around the estimated difference was high, the study had a very small sample size (n=55) and the authors did not specify whether they included women with pre-gestational or gestational diabetes.[22]

Strengths and limitations

A major strength of this study is its prospective design. Enrollment of study participants and collection of baseline characteristics took place close to the moment of GDM diagnosis and ahead of the CIMT outcome assessment. This implies that the choice of participation in the study is unlikely to be related to both the exposure and the outcome, which makes selection bias due to enrollment unlikely. Further, GDM was diagnosed using the new criteria of the IADPG. These criteria were derived based on the risk of adverse neonatal outcomes, such as birth weight, cord blood C-peptide levels, or percent infant body fat > 90th percentile.[15] They were endorsed by the World Health Organization along with several other bodies to achieve a universal consensus for GDM diagnosis and increase comparability of the evidence.[23,24] Another strength is the assessment of ultrasound CIMT using automated methods with manual tracing

For peer review only

For peer review only - http://bmjopen.bmj.com/site/about/guidelines.xhtml

adjustment, in accordance with the current guidelines in children.[16,25] The semi-automated methods are associated with a lower interoperator variability and high reliability,[16,25] including in infants, as it was previously proved in our laboratory.[12]

This study has some limitations. Firstly, our results have limited generalizability, as we used a convenient sample of pregnant women recruited from health care facilities in Switzerland. Secondly, the GDM glucose screening test (FPG or 75-g OGTT) varied between participants. This is because our hospital used a 2-step targeted approach for identifying women with GDM. While the 2-step approach is practical and more acceptable to patients,[26] it may be related to a lower likelihood of diagnosing GDM compared to a one-step universal screening based on a 75-g OGTT.[27] On the other hand, the IADGSP criteria, which have a lower threshold for a positive FPG test (≥5.1 mmol/L) compared to other guidelines,[23] may identify as having GDM women who are at low absolute risk for fetal and pregnancy complications and, thus, overdiagnose GDM in some populations.[28,29] Therefore, misclassification of the exposure cannot be excluded and our estimates of association might be biased, maybe underestimated. Additionally, women with GDM participated in *MySweetHeart* Trial and approximately half of them were assigned to a lifestyle and psychosocial intervention with the aim of improving their cardiometabolic outcomes. Although this intervention could have also modified the association of GDM with CIMT, this seems not likely, as mean CIMT values were very similar in offspring of women with GDM who participated in the intervention and the control arms of the trial. Thirdly, CIMT was assessed using conventional high-resolution ultrasound frequencies (< 15 MHz), which have a lower spatial resolution and, thus, tend to overestimate the arterial thickness in the young children when compared to very high-resolution ultrasound systems (25 to 55

For peer review only

MHz).[30,31] Measurement error in CIMT cannot be excluded, but systematic differences between the two groups are unlikely because the outcome assessors were blinded to the glycemic status of the mothers. Fourthly, while we adjusted for key confounders at the analysis stage, there is a possibility of bias due to unmeasured factors, such as family history of premature cardiovascular death, or residual confounding due to the relatively small sample size and imprecision in the measurement of confounder variables, especially in those self-reported. Lastly, our study was limited to CIMT, which is a measure of arterial structure. In fact, changes in the vessel function might occur earlier than changes in the vessel structure, therefore, a combination of vascular measures would be needed for a clearer view on the cardiovascular status of children exposed to adverse experiences in early life. However, certain techniques to assess arterial function and stiffness, such as flow-mediated dilation and pulse-wave velocity, are not currently feasible in the very young due to limited compliance and technical inconveniences.[18]

Implications and future research

Our results suggest that intrauterine exposure to GDM does not induce changes in the carotid artery structure that are detectable with conventional ultrasound techniques at birth and may not be linked to early vascular aging at this arterial site in the short term. Measurements at other arterial sites, such as the aorta,[32] may be more useful to investigate early or subtle abnormalities related to accelerated vascular aging or subclinical atherosclerosis. A long-term follow-up that includes complementary vascular measures, for instance, endothelium-dependent and endothelium-independent vasodilation or large-artery stiffness,[20] may shed further light on the cardiovascular health of children born to mothers with GDM.

For peer review only - http://bmjopen.bmj.com/site/about/guidelines.xhtml

For peer review only

Patient and public involvement

There was no patient or public involvement in the design, conduct, analysis, or reporting of this study's findings.

Author affiliations

[1]Population Health Laboratory (#PopHealthLab), University of Fribourg, Fribourg, Switzerland;
[2]Department of Epidemiology and Health Services, Center for Primary Care and Public Health (UNISANTÉ), University of Lausanne, Lausanne, Switzerland;
[3]Paediatric Cardiology Unit, Woman-Mother-Child Department, Lausanne University Hospital (CHUV), Lausanne, Switzerland;
[4]Institute of Primary Health Care (BIHAM), University of Bern, Bern, Switzerland;
[5]School of Global and Population Health, McGill University, Montréal, Canada

Authors' contributions

AC, NS, SDB, and YM designed the study and the data collection procedures with input from SEY, AME. SEY and AME collected baseline characteristics for participants without GDM. SDB and NS collected neonatal cardiovascular characteristics for all participants. SEY performed data management and curation. AME carried out the statistical analyses with input and supervision from AC. AME wrote the first draft of the manuscript with input from AC and NS. SDB, SEY, YM made critical revisions to the manuscript for important intellectual content. All authors read and approved the content of the manuscript.

For peer review only

Acknowledgements

The study teams of *MySweetHeart* Cohort and *MySweetHeart* Trial form *MySweetHeart* Research group and collaborated on data collection, management, and curation. We thank the following members of *MySweetHeart* Research Group, listed in alphabetical order: Amar Arhab, Pascal Bovet, Arnaud Chiolero, Stefano Di Bernardo, Adina Mihaela Epure, Sandrine Estoppey Younes, Leah Gilbert, Justine Gross, Antje Horsch, Stefano Lanzi, Seyda Mayerat, Yvan Mivelaz, Jardena J. Puder, Dan Quansah, Jean-Benoit Rossel, Nicole Sekarski, Umberto Simeoni, Bobby Stuijfzand, Yvan Vial.

Funding

This study was funded by the Swiss National Science Foundation (www.snf.ch; *MySweetHeart* Cohort project number 32003B-163240; *MySweetHeart* Trial project number 32003B_176119). The funder had no role in the study design, data collection and analysis, or interpretation of results.

Competing interests

None declared.

Consent for publication

Not applicable.

Ethics approval

Ethical approval was obtained through the Ethics Committee for Human Research of the Canton of Vaud (2016–00745).

For peer review only

For peer review only - http://bmjopen.bmj.com/site/about/guidelines.xhtml

Data availability statement

Data could be made available by the principal investigator and corresponding author (Prof Nicole Sekarski: nicole.sekarski@chuv.ch) on reasonable request.

For peer review only

For peer review only - http://bmjopen.bmj.com/site/about/guidelines.xhtml

REFERENCES

1. Reece EA, Leguizamon G, Wiznitzer A. Gestational diabetes: the need for a common ground. *Lancet* 2009;373(9677):1789-97. doi: 10.1016/s0140-6736(09)60515-8.

2. Reece EA. The fetal and maternal consequences of gestational diabetes mellitus. *J Matern Fetal Neonatal Med* 2010;23(3):199-203. doi: 10.3109/14767050903550659.

3. American Diabetes Association (ADA). 2. Classification and Diagnosis of Diabetes: Standards of Medical Care in Diabetes—2021. *Diabetes Care* 2021;44(Supplement 1):S15-S33. doi: 10.2337/dc21-S002.

4. International Diabetes Federation. IDF Diabetes Atlas. 9th edition. Brussels, Belgium: International Diabetes Federation, 2019.

5. Damm P, Houshmand-Oeregaard A, Kelstrup L, et al. Gestational diabetes mellitus and long-term consequences for mother and offspring: a view from Denmark. *Diabetologia* 2016;59(7):1396-99. doi: 10.1007/s00125-016-3985-5.

6. Li JW, He SY, Liu P, et al. Association of gestational diabetes mellitus (GDM) with subclinical atherosclerosis: a systemic review and meta-analysis. *BMC Cardiovasc Disord* 2014;14:132. doi: 10.1186/1471-2261-14-132.

7. Tobias DK, Stuart JJ, Li S, et al. Association of History of Gestational Diabetes With Long-term Cardiovascular Disease Risk in a Large Prospective Cohort of US Women. *JAMA Intern Med* 2017;177(12):1735-42. doi: 10.1001/jamainternmed.2017.2790.

8. Epure AM, Rios-Leyvraz M, Anker D, et al. Risk factors during first 1,000 days of life for carotid intima-media thickness in infants, children, and adolescents: A

For peer review only - http://bmjopen.bmj.com/site/about/guidelines.xhtml

systematic review with meta-analyses. *PLoS Med* 2020;17(11):e1003414. doi: 10.1371/journal.pmed.1003414.

9. Giannopoulou EZ, Doundoulakis I, Antza C, et al. Subclinical arterial damage in children and adolescents with type 1 diabetes: A systematic review and meta-analysis. *Pediatr Diabetes* 2019;20(6):668-77. doi: 10.1111/pedi.12874.

10. Hanson MA, Gluckman PD. Early developmental conditioning of later health and disease: physiology or pathophysiology? *Physiol Rev* 2014;94(4):1027-76. doi: 10.1152/physrev.00029.2013.

11. Aceti A, Santhakumaran S, Logan KM, et al. The diabetic pregnancy and offspring blood pressure in childhood: a systematic review and meta-analysis. *Diabetologia* 2012;55(11):3114-27. doi: 10.1007/s00125-012-2689-8.

12. Mivelaz Y, Di Bernardo S, Boulos Ksontini T, et al. Feasibility and reliability of carotid intima-media thickness measurements in nonsedated infants. *J Hypertens* 2016;34(11):2227-32. doi: 10.1097/hjh.0000000000001065.

13. Di Bernardo S, Mivelaz Y, Epure AM, et al. Assessing the consequences of gestational diabetes mellitus on offspring's cardiovascular health: MySweetHeart Cohort study protocol, Switzerland. *BMJ Open* 2017;7(11):e016972. doi: 10.1136/bmjopen-2017-016972.

14. Horsch A, Gilbert L, Lanzi S, et al. Improving cardiometabolic and mental health in women with gestational diabetes mellitus and their offspring: study protocol for MySweetHeart Trial, a randomised controlled trial. *BMJ Open* 2018;8(2):e020462. doi: 10.1136/bmjopen-2017-020462.

15. Metzger BE, Gabbe SG, Persson B, et al. International association of diabetes and pregnancy study groups recommendations on the diagnosis and classification

For peer review only

of hyperglycemia in pregnancy. *Diabetes Care* 2010;33(3):676-82. doi: 10.2337/dc09-1848.

16. Urbina EM, Williams RV, Alpert BS, et al. Noninvasive assessment of subclinical atherosclerosis in children and adolescents: recommendations for standard assessment for clinical research: a scientific statement from the American Heart Association. *Hypertension* 2009;54(5):919-50. doi: 10.1161/hypertensionaha.109.192639.

17. Mosteller RD. Simplified calculation of body-surface area. *N Engl J Med* 1987;317(17):1098. doi: 10.1056/nejm198710223171717.

18. Torigoe T, Dallaire F, Slorach C, et al. New Comprehensive Reference Values for Arterial Vascular Parameters in Children. *J Am Soc Echocardiogr* 2020 doi: 10.1016/j.echo.2020.03.001.

19. Sarkola T, Manlhiot C, Slorach C, et al. Evolution of the arterial structure and function from infancy to adolescence is related to anthropometric and blood pressure changes. *Circulation* 2012;126(21).

20. Sundholm JKM, Litwin L, Rönö K, et al. Maternal obesity and gestational diabetes: Impact on arterial wall layer thickness and stiffness in early childhood - RADIEL study six-year follow-up. *Atherosclerosis* 2019 doi: 10.1016/j.atherosclerosis.2019.01.037.

21. Ayer JG, Harmer JA, Nakhla S, et al. HDL-cholesterol, blood pressure, and asymmetric dimethylarginine are significantly associated with arterial wall thickness in children. *Arterioscler Thromb Vasc Biol* 2009;29(6):943-49. doi: 10.1161/ATVBAHA.109.184184.

For peer review only - http://bmjopen.bmj.com/site/about/guidelines.xhtml

22. Atabek ME, Çağan HH, Eklioğlu BS, et al. Absence of increase in carotid artery Intima-Media thickness in infants of diabetic mothers. *J Clin Res Pediatr Endocrinol* 2011;3(3):144-48. doi: 10.4274/jcrpe.v3i3.28.

23. World Health Organization. Diagnostic criteria and classification of hyperglycaemia first detected in pregnancy: a World Health Organization Guideline. *Diabetes Res Clin Pract* 2014;103(3):341-63. doi: 10.1016/j.diabres.2013.10.012.

24. Yuen L, Saeedi P, Riaz M, et al. Projections of the prevalence of hyperglycaemia in pregnancy in 2019 and beyond: Results from the International Diabetes Federation Diabetes Atlas, 9th edition. *Diabetes Res Clin Pract* 2019;157:107841. doi: 10.1016/j.diabres.2019.107841.

25. Dalla Pozza R, Ehringer-Schetitska D, Fritsch P, et al. Intima media thickness measurement in children: A statement from the Association for European Paediatric Cardiology (AEPC) Working Group on Cardiovascular Prevention endorsed by the Association for European Paediatric Cardiology. *Atherosclerosis* 2015;238(2):380-7. doi: 10.1016/j.atherosclerosis.2014.12.029.

26. Agarwal MM, Dhatt GS, Shah SM. Gestational Diabetes Mellitus: Simplifying the International Association of Diabetes and Pregnancy diagnostic algorithm using fasting plasma glucose. *Diabetes Care* 2010;33(9):2018. doi: 10.2337/dc10-0572.

27. Mahdavian M, Hivert M-F, Baillargeon J-P, et al. Gestational Diabetes Mellitus: Simplifying the International Association of Diabetes and Pregnancy Diagnostic Algorithm Using Fasting Plasma Glucose. Comment on Agarwal, Dhatt, and Shah. *Diabetes Care* 2010;33(11):e145. doi: 10.2337/dc10-1454.

For peer review only

28. McIntyre HD, Jensen DM, Jensen RC, et al. Gestational Diabetes Mellitus: Does One Size Fit All? A Challenge to Uniform Worldwide Diagnostic Thresholds. *Diabetes Care* 2018;41(7):1339-42. doi: 10.2337/dc17-2393.

29. Brodersen J, Schwartz LM, Heneghan C, et al. Overdiagnosis: what it is and what it isn't. *BMJ Evidence-Based Medicine* 2018;23(1):1. doi: 10.1136/ebmed-2017-110886.

30. Olander RFW, Sundholm JKM, Ojala TH, et al. Neonatal Arterial Morphology Is Related to Body Size in Abnormal Human Fetal Growth. *Circ Cardiovasc Imaging* 2016;9(9) doi: 10.1161/CIRCIMAGING.116.004657.

31. Sarkola T, Slorach C, Hui W, et al. Transcutaneous very-high resolution ultrasound for the quantification of carotid arterial intima-media thickness in children - feasibility and comparison with conventional high resolution vascular ultrasound imaging. *Atherosclerosis* 2012;224(1):102-7. doi: 10.1016/j.atherosclerosis.2012.06.054.

32. Koklu E, Akcakus M, Kurtoglu S, et al. Aortic intima-media thickness and lipid profile in macrosomic newborns. *Eur J Pediatr* 2007;166(4):333-8. doi: 10.1007/s00431-006-0243-8.

For peer review only

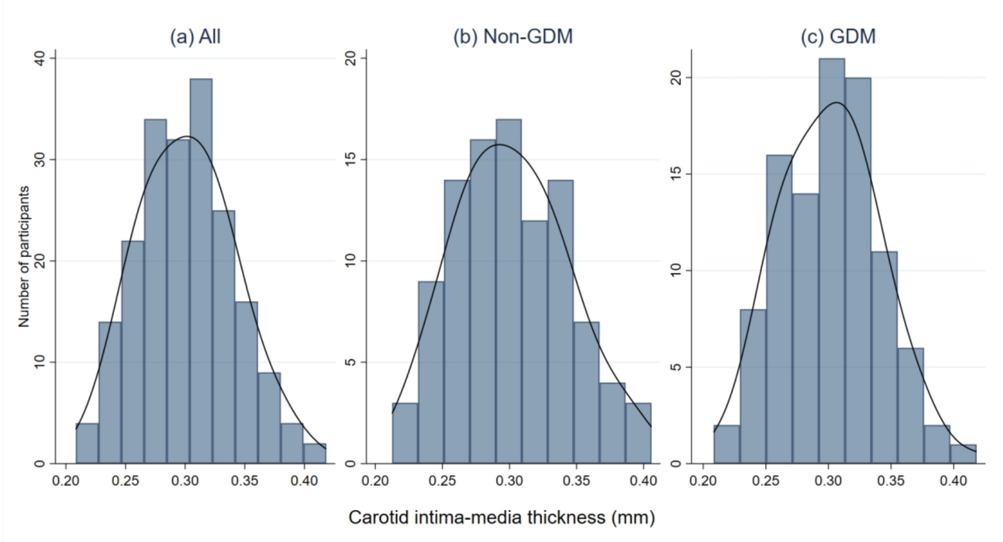

This figure shows the distribution of CIMT values in our sample, overall (n=200) and by GDM exposure (Non-GDM: n=99; GDM: n=101). The black line represents the kernel density estimate. Abbreviations: CIMT, carotid intima-media thickness; GDM, gestational diabetes mellitus.

599x326mm (72 x 72 DPI)

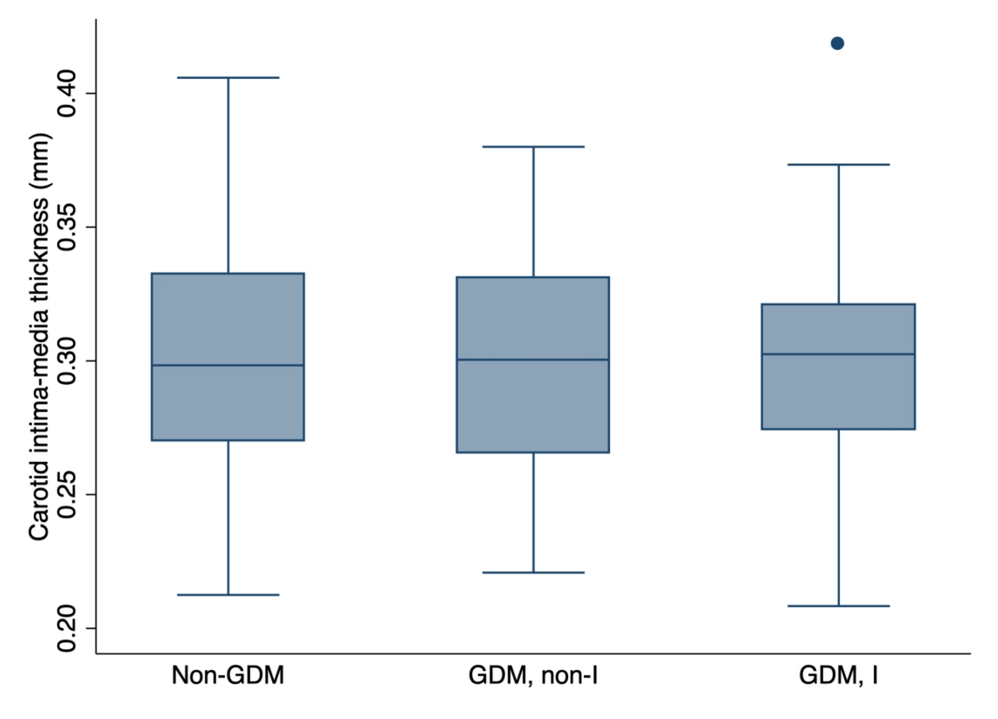

This figure shows the distribution of CIMT in the offspring of women without GDM (Non-GDM; n=99) and the offspring of women with GDM who were assigned to no intervention (GDM, non-I; n=48) or to a lifestyle and psychosocial intervention (GDM, I; n=53) as part of their participation in the MySweetHeart Trial. The line inside the box represents the median value of the distribution, while the lower and upper boundaries of the box represent the first (Q1) and third quartiles (Q3), respectively. The interquartile range (IQR) corresponds to Q3 – Q1. The whiskers extend from either side of the box up to 1.5*IQR (i.e., Q1-1.5*IQR and Q3+1.5*IQR).

378x275mm (144 x 144 DPI)

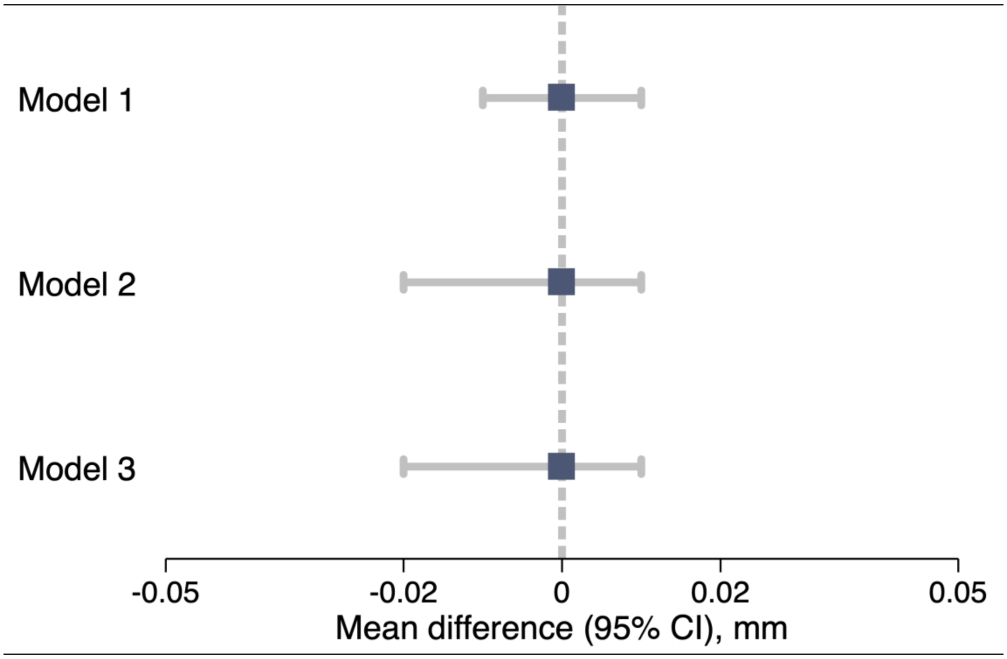

The boxes represent the mean differences in CIMT between offspring of women with and without GDM (i.e., GDM versus non-GDM). The horizontal lines represent the 95% CIs. The plot was constructed using regression estimates and models presented in Table 2. Model specification: Model 1 is unadjusted, while Models 2 and 3 are adjusted for various factors as described in the methods and footnote of Table 2. Abbreviations: CI, confidence interval; CIMT, carotid intima-media thickness; GDM, gestational diabetes mellitus.

423x275mm (144 x 144 DPI)

SUPPLEMENTARY MATERIAL

Gestational diabetes mellitus and offspring's carotid intima-media thickness at birth: *MySweetHeart* Cohort Study

Adina Mihaela Epure[1,2], Stefano Di Bernardo[3], Yvan Mivelaz[3], Sandrine Estoppey Younes[2], Arnaud Chiolero[1,4,5¶], Nicole Sekarski[3¶], on behalf of *MySweetHeart* Research Group

[1]Population Health Laboratory (#PopHealthLab), University of Fribourg, Fribourg, Switzerland;

[2]Department of Epidemiology and Health Services, Center for Primary Care and Public Health (UNISANTÉ), University of Lausanne, Lausanne, Switzerland;

[3]Paediatric Cardiology Unit, Woman-Mother-Child Department, Lausanne University Hospital (CHUV), Lausanne, Switzerland;

[4]Institute of Primary Health Care (BIHAM), University of Bern, Bern, Switzerland;

[5]School of Global and Population Health, McGill University, Montréal, Canada

¶ These authors contributed equally to this work.

For peer review only - http://bmjopen.bmj.com/site/about/guidelines.xhtml

Table S1 The relationship of GDM and assignment or not to a lifestyle and psychosocial intervention with offspring's CIMT at birth.

	Mean (sd), mm	**Model 1 (n=200)**		**Model 2 (n=165)**		**Model 3 (n=165)**	
		Mean difference (95% CI), mm	p	Mean difference (95% CI), mm	p	Mean difference (95% CI), mm	p
Non-GDM	0.30 (0.04)	ref	ref	ref	ref	ref	ref
GDM, non-I	0.30 (0.04)	0.00 (-0.01 to 0.01)	0.94	0.00 (-0.02 to 0.02)	0.93	0.00 (-0.02 to 0.01)	0.91
GDM, I	0.30 (0.04)	0.00 (-0.01 to 0.01)	0.98	-0.01 (-0.03 to 0.01)	0.19	-0.01 (-0.02 to 0.01)	0.25

Estimates were obtained from linear regression models with the following specification: Model 1: unadjusted estimates; Model 2: estimates adjusted for maternal pre-pregnancy BMI, education, and tobacco smoking; offspring family history of diabetes and sex; Model 3: estimates adjusted for maternal pre-pregnancy BMI, education, and tobacco smoking; offspring family history of diabetes, sex, body surface area and age at CIMT assessment. The outcome variable (i.e., CIMT) was continuous. The exposure variable was categorical with 3 levels (Non-GDM/ GDM, non-I/ GDM, I; the reference category was Non-GDM). Similar results were obtained when Model 1 was run in the sample (n=165) with data on outcome, exposure, and all covariates included in Models 2 and 3 (GDM, non-I: 0.00 mm (95% CI: -0.01 to 0.01; p=0.99); GDM, I: -0.01 mm (95% CI: -0.02 to 0.01; p=0.29)). Abbreviations: CI, confidence interval; CIMT, carotid intima-media thickness; GDM, gestational diabetes mellitus; I, intervention; n, total number of participants; p, p-value; sd, standard deviation; ref, reference group.

For peer review only

Fig. S1 Illustration of the relationship of GDM and assignment or not to a lifestyle and psychosocial intervention with offspring's CIMT through a forest plot.

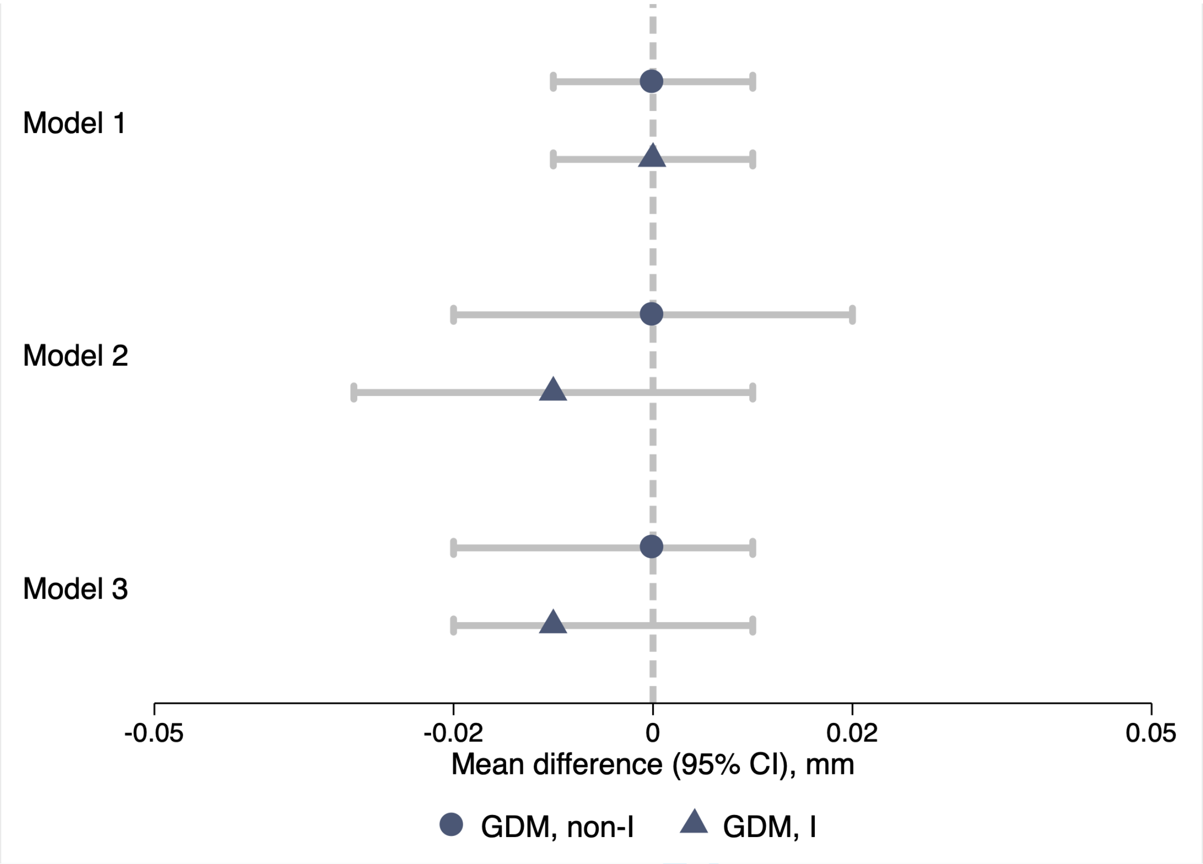

Figure legend The circles represent mean differences in CIMT between offspring of women with GDM assigned to no intervention (*GDM, non-I*) and offspring of women without GDM (*Non-GDM*). The triangles represent mean differences in CIMT between offspring of women with GDM assigned to a lifestyle and psychosocial intervention (*GDM, I*) and offspring of women without GDM (*Non-GDM*). The horizontal lines represent the 95% CIs. The plot was constructed using regression estimates and models presented in Table S1. Model specification: Model 1 is unadjusted, while Models 2 and 3 are adjusted for various factors as described in the methods and footnote of Table S1. Abbreviations: CI, confidence interval; CIMT, carotid intima-media thickness; GDM, gestational diabetes; I, intervention.

For peer review only - http://bmjopen.bmj.com/site/about/guidelines.xhtml

Fig. S2 Dot plot of CIMT at birth by gestational diabetes mellitus (GDM) and assignment to a lifestyle and psychosocial intervention (I).

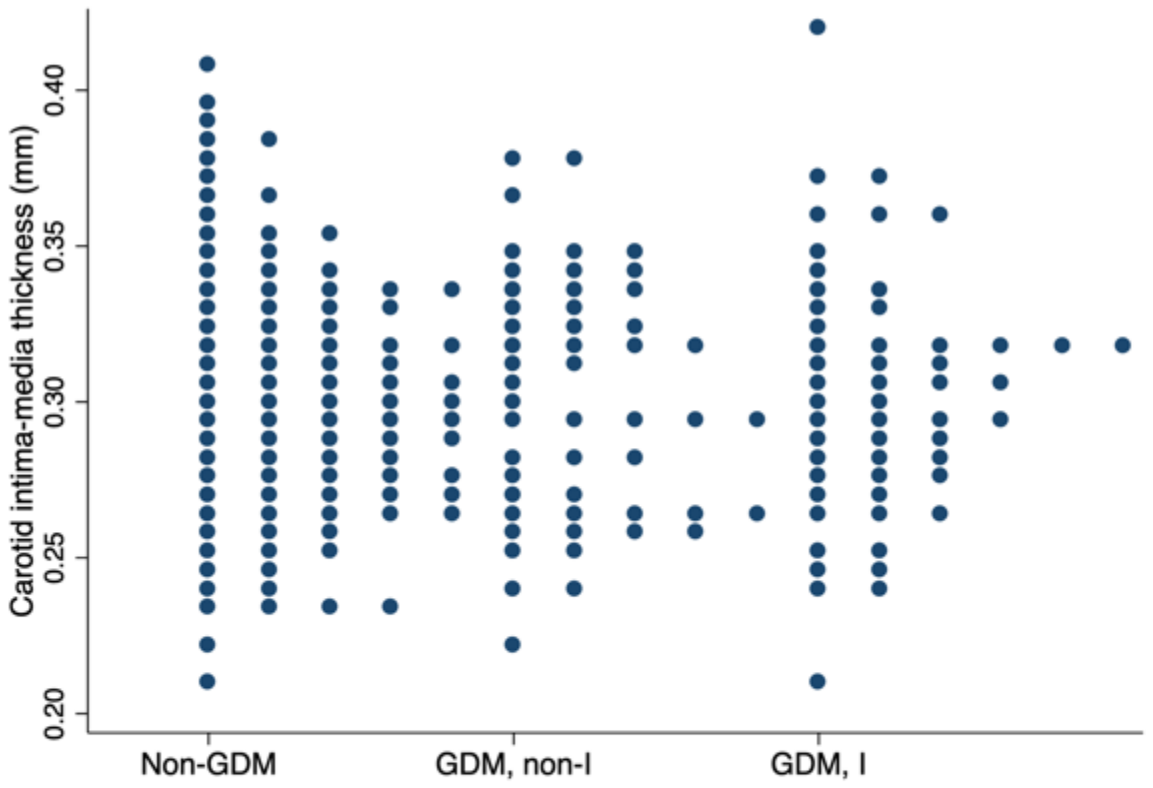

Figure legend This figure shows the distribution of CIMT in the offspring of women without GDM (*Non-GDM*) and the offspring of women with GDM who were assigned to no intervention (*GDM, non-I*) or to a lifestyle and psychosocial intervention (*GDM, I*) as part of their participation in the *MySweetHeart* Trial. Abbreviations: CIMT, carotid intima-media thickness; GDM, gestational diabetes mellitus; I, intervention.

For peer review only - http://bmjopen.bmj.com/site/about/guidelines.xhtml
